# Artificial intelligence can match domain experts in evidence extraction and critical appraisal of microbial oncogenesis research publications

**DOI:** 10.3389/fcimb.2026.1876326

**Published:** 2026-08-06

**Authors:** Kaela Kokkas, Hairong Wang, Richard Klein, Nazir A. Ismail, Natalie Irwin, Mohammad Z. Moonsamy, Kubendran Naidoo, Jeremy Nel, Ekene E. Nweke, Raveen Parboosing, Emmanuel K. Sekyi, Rebecca T. van Dorsten, Bruce A. Bassett, Robert F. Breiman

**Affiliations:** 1 Department of Clinical Microbiology and Infectious Diseases, Faculty of Health Sciences, University of the Witwatersrand, Johannesburg, South Africa; 2 School of Computer Science and Applied Mathematics, University of the Witwatersrand, Johannesburg, South Africa; 3 Wits Machine Intelligence and Neural Discovery (MIND) Institute, University of the Witwatersrand, Johannesburg, South Africa; 4 Department of Clinical Microbiology and Infectious Diseases, National Health Laboratory Service and Faculty of Health Sciences, University of Witwatersrand, Johannesburg, South Africa; 5 Division of Medical Oncology, Department of Internal Medicine, University of the Witwatersrand, Johannesburg, South Africa; 6 Infectious Diseases and Oncology Research Institute (IDORI), Faculty of Health Sciences, University of the Witwatersrand, Johannesburg, South Africa; 7 South African Medical Research Council Vaccines and Infectious Diseases Analytics Research Unit, Faculty of Health Sciences, University of the Witwatersrand, Johannesburg, South Africa; 8 South African Medical Research Council Wits Antiviral Gene Therapy Research Unit, Faculty of Health Sciences, University of the Witwatersrand, Johannesburg, South Africa; 9 Department of Global Health, Rollins School of Public Health, Emory University, Atlanta, GA, United States; 10 National Health Laboratory Service, Johannesburg, South Africa; 11 Department of Molecular Medicine and Haematology, School of Pathology, University of the Witwatersrand, Johannesburg, South Africa; 12 Wits Research Institute for Malaria, Faculty of Health Sciences, National Health Laboratory Service, University of the Witwatersrand, Johannesburg, South Africa; 13 Division of Infectious Diseases, School of Clinical Medicine, Faculty of Health Sciences, University of the Witwatersrand, Johannesburg, South Africa; 14 Department of Surgery, Faculty of Health Sciences, University of Witwatersrand, Johannesburg, South Africa; 15 Division of Virology, University of Witwatersrand National Health Laboratory Service, Johannesburg, South Africa; 16 OncoVectra, London, United Kingdom

**Keywords:** large language models, artificial intelligence, critical appraisal, evidence extraction, evidence synthesis, evidence evaluation, biomedical literature, microbial oncogenesis

## Abstract

**Background:**

Confirmed oncogenic microbes contribute significantly to cancer burden. Identifying and confirming novel microbial oncogenicity could yield strategies and tools that will reduce disease burdens. However, relevant evidence may be dispersed across a vast biomedical literature that is infeasible for humans to comprehensively synthesize. Large Language Models (LLMs) may enable scalable, expert-level systematic evidence synthesis to identify high priority microbe-cancer pairs; however, such capabilities have not yet been demonstrated.

**Methods:**

Domain experts were recruited to create a human-validated test dataset to benchmark the performance of LLMs (Gemini 2.5 Pro, Gemini 2.5 Flash, GPT-5, and GPT-5 Nano) on 24 original research papers using Mouse Mammary Tumor Virus-Like Virus and breast cancer as a case study. We devised a structured template for evidence extraction and appraisal of papers, consisting of multiple choice, Likert-scale, multi-select, and free-text question types (77 question items across 24 papers). Agreement between (1) experts, and (2) experts and each LLM, was determined per question instance using novel scoring metrics. LLMs were assessed by comparing inter-expert and expert-LLM agreement score distributions to determine whether LLMs behaved as additional experts by either increasing or maintaining inter-expert agreement. Free-text responses were further evaluated qualitatively.

**Results:**

Across all question types, LLM responses aligned closely with expert assessments, with two models (GPT-5, GPT-5 Nano) achieving score distributions statistically indistinguishable from those of experts. Gemini models behaved similarly for most tasks but were significantly more lenient in applying microbial oncogenesis criteria, often over-attributing criteria fulfillment. Hallucinations were rare, although more frequent in smaller models (Gemini 2.5 Flash, GPT-5 Nano). Methodological appraisal and identification of contradictions within full-text papers were the most persistent areas of LLM vulnerability, however, the error rate could not be directly compared with experts.

**Conclusions:**

Two LLMs (GPT-5, GPT-5 Nano) were indistinguishable from domain experts on structured domain research paper evaluation tasks. This evidence supports use of LLMs for automated systematic evidence synthesis. However, methodological appraisal tasks and contradiction identification in full-text papers remain weaknesses requiring further investigation, strengthening, and possibly multi-model strategies.

## 1 Introduction

The identification of human papillomavirus (HPV) as the cause of cervical cancer (Watts, 2023) established cancer as a preventable disease through vaccination, screening, and treatment of infection (World Health Organization (WHO), 2022). Since then, 11 microbial Group 1 carcinogens (refers to those agents with sufficient evidence of carcinogenicity in humans) have been identified (IARC Working Group on the Evaluation of Carcinogenic Risks to Humans, 2012; International Agency for Research on Cancer (IARC), 2025), with infectious agents linked to at least 20% of cancer cases worldwide and 30% of cases in sub-Saharan Africa (de Martel et al., 2020; Parkin et al., 2020). The identification of additional oncogenic microbes could provide opportunities to significantly reduce cancer incidence, morbidity, and mortality via development and integration of targeted interventions into public health policies.

However, identifying the most probable and impactful microbe-cancer pairs (MCPs) for further research is a significant challenge, with over 1400 possible microbial species (Microbiology by numbers, 2011), more than 100 cancer types (Institute, N. C, [[NoYear]]; National Cancer Institute (NCI), 2021), and variability in oncogenic mechanisms, the microbial types, and their molecular structures which could impact oncogenicity (Moore and Chang, 2010). This has resulted in a division of efforts with multiple MCPs being investigated simultaneously (Luczynski et al., 2022; Miyabayashi et al., 2022; Zerdan et al., 2022; Bernardo et al., 2023; Dougherty and Jobin, 2023). While the existing literature exploring MCPs is not exhaustive, there are many proposed pairs with compelling evidence that warrant further investigation. Identifying a starting point requires a comprehensive, systematic evidence synthesis process to determine which MCPs offer both the greatest plausibility and public health benefit. However, the existing literature and one million articles published yearly in more than 13000 active indexed biomedical science journals (Ghasemi et al., 2022), renders a traditional “human-driven” literature search and evaluation infeasible, even after screening and filtering for relevance. Artificial Intelligence (AI) provides a potential for augmenting traditional literature search strategies with tools capable of rapidly evaluating and synthesizing available evidence.

AI, especially in the form of Large Language Models (LLMs), is increasingly demonstrating value for strengthening and accelerating a variety of facets of medical research, from reviewing and evaluating medical data (Bülbül et al., 2025; Brigo et al., 2025; Gottweis et al., 2025; Koh et al., 2025), drug discovery (Zhang et al., 2025), predicting and designing protein folding structures (Jumper et al., 2021; Zambaldi et al., 2024), to multi-use research assistants (Tsafnat et al., 2014; van de Schoot et al., 2021; Chappell et al., 2023; Huang et al., 2025; Li et al., 2025; Nordmann et al., 2025), and specialized systemic review tools. Recent advancements in accessibility, affordability, and the capability of pre-trained LLMs, including the advent of reasoning LLMs capable of solving complex reasoning tasks, make analyzing the vast scientific literature potentially feasible. However, concerns regarding the trustworthiness of these tools necessitate validation (McFadden et al., 2023). These concerns include: (1) LLMs’ tendency to hallucinate and produce plausibly sounding yet factually incorrect outputs (Kim et al., 2025), and (2) limited assessments of the validity of outputs complicated by the “black-box problem” where neural networks’ rationale and critical thinking processes are indiscernible (Castelvecchi, 2016). Thus, to allow for further integration of these systems into current research workflows, we need to ensure that models’ outputs are consistently trustworthy, accurate, and controllable when performing these tasks.

**Figure 1:**
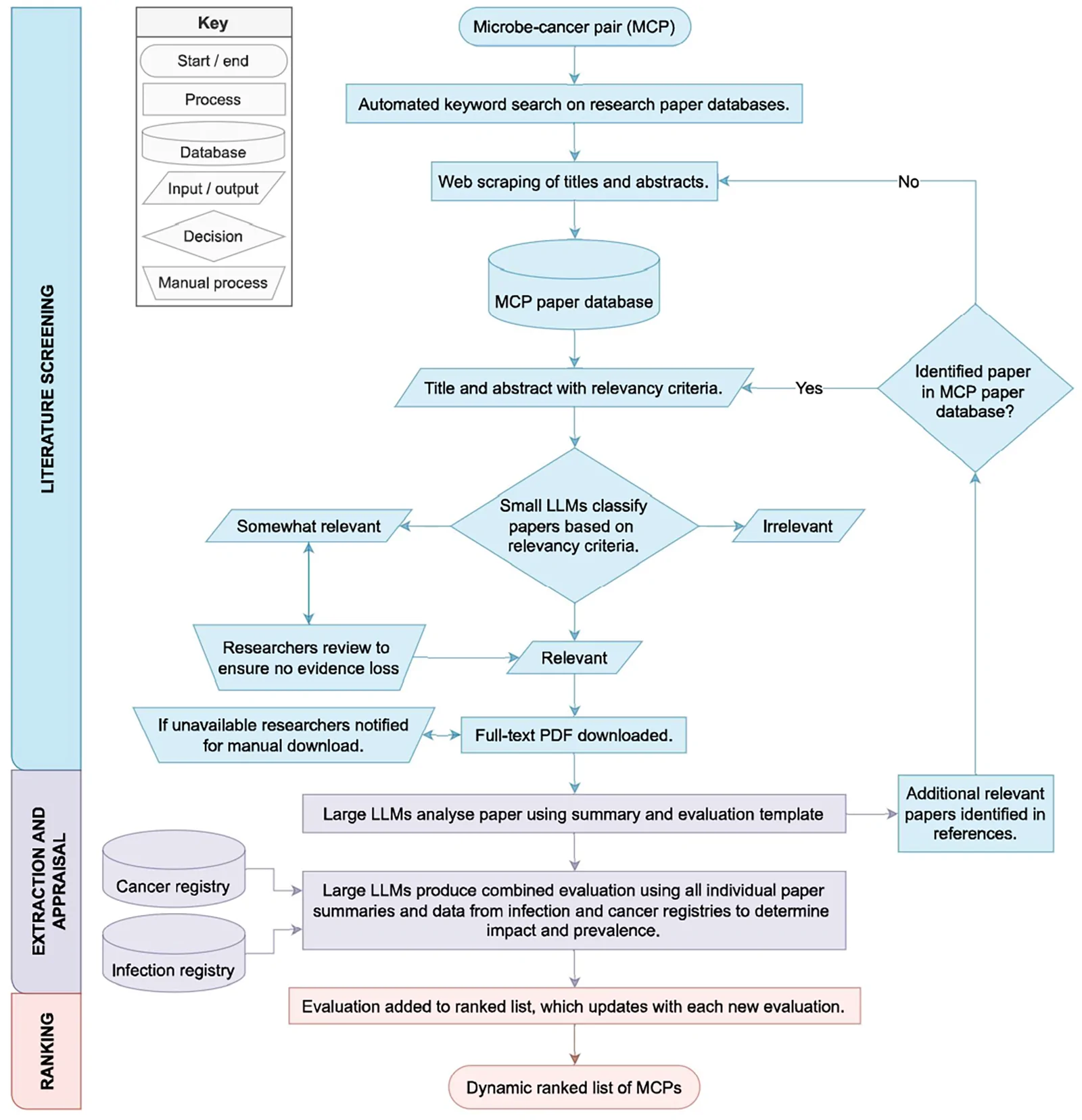
Overview of proposed AI system. Initial keyword search is performed for microbe-cancer pairs (MCPs) on research databases, with web scraping and classification of titles and abstracts by small LLMs using pre-defined relevancy criteria. Papers classified as *Somewhat relevant* may be manually reviewed or included in next steps if limited papers retrieved. Full-text versions of *Relevant* papers are downloaded and analyzed by LLMs using extraction template. Potentially *Relevant* papers in references are cross-checked with paper storage database and searched for if absent. LLMs produce final MCP evaluation using individual paper summaries and data from infection and cancer registries to determine geographical impact and prevalence of both the cancer and the microbe (when results are available and accessible). This final MCP evaluation is then added to a dynamic list of MCPs, ranked based on a standardized scoring matrix including factors such as potential impact if targeted, confidence in causal relationship, and novelty.

We envision using AI to filter and analyze literature on MCPs, ultimately producing a ranked list based on a standardized scoring matrix for prioritizing investigations (**Figure 1**). To begin validating this proposed method, we initially set up an automated search to retrieve papers from biomedical databases and assessed whether small LLMs could accurately screen these papers using nuanced classification criteria (Dawood et al., 2025). In this paper, we evaluate and compare pre-trained reasoning LLMs’ abilities to analyze and evaluate individual research papers using an extraction template (questionnaire), and benchmark four LLMs’ assessments (Gemini 2.5 Pro, Gemini 2.5 Flash, GPT-5, and GPT-5 Nano) with that of a panel of experts versed in biomedical science. One specific potential MCP was used for this validation: Human Mammary Tumor Virus (HMTV)/Mouse Mammary Tumor Virus-Like Virus (MMTV-LV) and breast cancer. Our objectives were to: (1) determine whether LLMs could interpret nuanced biomedical language, (2) critically appraise research, (3) apply causal criteria to expert standards, and (4) produce accurate, trustworthy outputs with minimal hallucinations or omissions, and ultimately utilize our findings to highlight opportunities for improvement, with potential applicability across other biomedical research domains.

## 2 Methods

### 2.1 Extraction template development

Rigorous evidence syntheses require transparent and reproducible methodology, including details of how studies are appraised and analyzed (Gough et al., 2020). We developed a structured, standardized extraction template (questionnaire) to ensure repeatable and thorough information extraction. The template was designed to: (1) control and increase transparency of LLM outputs by documenting processes and reasoning, (2) improve reasoning via chain-of-thought (CoT) prompting (Wei et al., 2022), (3) ensure consideration of factors beyond each paper’s stated primary results, and (4) enable comparison between human and LLM responses. The template consisted of pre-prompt context describing the LLM’s role and six question sections addressing different aspects of the paper, namely *“Relevancy”*, *“Paper Bibliographic Details”*, *“Paper Integrity and Reliability”*, *“Summary of Paper Contents”*, *“Strength of Evidence”*, and *“Microbial Oncogenesis Criteria”* (see **Table 1** for more details on each section).

**Table 1:** Overview of the contents of each question section in the extraction template.

Extraction template question sections and their contents	
Question section	Contents
Relevancy	Relevancy classification criteria followed by questions asking for a classification of the paper (MCQ) and an explanation of why (long-answer).
Paper Bibliographic Details	Questions for details to trace papers, including the title, publication date, journal of publication, study design, country of origin, and DOI.
Paper Integrity and Reliability	Questions to determine the reliability of the paper, including whether references were appropriate, and if there was any evidence of a conflict of interest, data or image manipulation, or any contradictions, with a final overall appraisal via a paper reliability score (Likert scale) from 1-5.
Summary of Paper Contents	Questions for more details of the paper, including the aim and hypothesis, sample characteristics, whether the study’s findings are applicable to African populations, and important methodological factors of the study.
Strength of Evidence	Questions to determine the strength of the paper’s evidence, including how data was collected, the appropriateness of the sample size, whether statistical analysis was appropriate, if there were any errors in analysis or presentation of results, and whether there are any limitations to using the paper’s findings as evidence for or against the MCP, with a final question asking for an overall appraisal via a strength of evidence score (Likert scale) from 1-5.
Microbial Oncogenesis Criteria	Descriptions of each criterion followed by questions on whether the paper fulfilled, refuted, left the criterion uncertain, or did not examine the criterion, as well as why (e.g., “Appropriate methodology/tools”, “Insufficient power”), and to extract the evidence for or against the criterion from the paper. The values for each of the criterions (1 for fulfilled, -1 for refuted, and 0 for either not investigated or left uncertain) are summed to produce the cumulative oncogenesis score (0-10, treated as a Likert scale), with a final question asking for a rating of the paper’s impact on the MCP’s plausibility (Likert scale) from 1-5

The template included different question types, with 22 multiple-choice questions (MCQs), four Likert scales, 10 multi-select questions, and 40 long-answer (free-text) questions. MCQs refer to questions where only one option can be selected, while multi-select questions refer to questions where any number of options can be selected concurrently. Completing all MCQs, Likert scales, and 14 specific long-answer questions was mandatory. Twenty-six long-answer questions and all 10 multi-select questions were conditional, based on the response to the previous question. Questions were iteratively refined prior to final experiments from team discussions and pilot sessions. The full extraction template can be found in the **Supplementary Material**.

### 2.2 Human-validated test dataset creation

#### 2.2.1 Locating papers

The MCP of HMTV/MMTV-LV and breast cancer was chosen for validation of the system for four reasons. First, its carcinogenicity in humans is unconfirmed, which reflects the intended use-case for our pipeline. Second, there is conflicting evidence for this MCP, enabling us to see how the LLMs would interpret and rate positive, negative, and neutral findings. Third, limited evidence for this MCP in the African context, which is a common challenge across many MCPs under investigation, provides an opportunity to evaluate how the system interprets and applies findings from foreign studies to Africa. Finally, the combination of human and animal research (mouse models) for this MCP allowed us to determine whether LLMs could interpret and apply findings from animal models to humans.

Twenty-four original research papers that could assist in determining whether HMTV/MMTV-LV causes breast cancer in humans, with differing methodologies and the type and strength of evidence provided, were selected from the PubMed database (Otten et al., 1988; Goedert et al., 2006; Indik et al., 2007; Callahan et al., 2012; Khalid et al., 2013; Kulkarni et al., 2013; Morales-Sanchez et al., 2013; Tabriz et al., 2013; Cedro-Tanda et al., 2014; Mazzanti et al., 2015; Reza et al., 2015; Naushad et al., 2017; Perzova et al., 2017; Shariatpanahi et al., 2017; Dossary et al., 2018; Kincaid et al., 2018; Naccarato et al., 2019; de Sousa Pereira et al., 2020; Lessi et al., 2020; Gupta et al., 2021; Gupta et al., 2022; Stewart and Chen, 2022; Khalid et al., 2023; James and Georgopoulos, 2024). These 24 papers were drawn from the classification dataset (*Relevant* category) (Dawood et al., 2025), consisting of an initial 20 that very clearly fulfilled the *Relevant* classification criteria, supplemented by four papers selected for their unique and nuanced study designs (i.e., where a *Relevant* article may initially seem unrelated to the topic, and require deeper biomedical knowledge to discern its potential effect on the MCP’s plausibility), enabling evaluation of LLM performance under a variety of conditions.

#### 2.2.2 Expert recruitment

The extraction template consists of two categories of analytical tasks: (1) questions requiring extensive domain-specific knowledge, and (2) extractive, rule-based, or statistical appraisal questions requiring formal research training but not domain specialization. Category one questions were answered by domain experts, with three experts per paper from a group of seven experts (RD, NI, NAI, KN, JN, EEN, and RP), all of whom were recruited from the University of the Witwatersrand and included specialist clinicians and scientists in oncology, clinical microbiology, pathology, infectious diseases, and virology. Category two questions, which were objective and/or extractive in nature and therefore only required one evaluator, were answered by a clinician-scientist trainee with training in data analytics and biostatistics (KK). These included extraction of bibliographic details of the paper (e.g., title, publication date, publishing journal), identifying contradictions (further evaluated during team discussions to ensure agreement), and bullet-point summaries of the study samples (see extraction template in **Supplementary Material** for questions in each category).

#### 2.2.3 Expert response collection

Author EKS created a web application for the purposes of this study (available at data.oncovectra.com) that served as a united interface for extraction and appraisal, with dual display of the extraction template questions and the research paper. Papers were uploaded to the platform and assigned to experts based on training specialty and research interest suitability, while ensuring three experts per paper. Experts received a video tutorial on how to use the platform. Experts were aware that the study aimed to compare LLM and domain expert responses, but were blinded to LLM outputs during annotation. The human-validated test dataset is available in the **Supplementary Material**.

### 2.3 LLM selection

We chose four LLMs across two leading LLM vendors: Google (Gemini 2.5 Pro (Comanici et al., 2025) and Gemini 2.5 Flash (Comanici et al., 2025)) and OpenAI (GPT-5 (Singh, 2026) and GPT-5 Nano (Singh, 2026)). This included two frontier reasoning models (Gemini 2.5 Pro and GPT-5) selected for their competitive performance on various reasoning and medical benchmarks at the time of model selection and two lightweight and much cheaper models (Gemini 2.5 Flash and GPT-5 Nano). All four selected LLMs had context-windows ≥128K to allow for input of the research paper and extraction template, and had multi-modal vision capability to facilitate interpretation of images and graphical data. Default parameters were used for all models, except for those related to thinking or reasoning effort, where the highest level was used. Initial outputs (using full versions of all 24 papers and the complete extraction template) were generated on 17 November 2025, using stable Gemini API versions gemini-2.5-pro and gemini-2.5-flash, and GPT-5 model API snapshots gpt-5-2025-08–07 and gpt-5-nano-2025-08-07. All subsequent experiments were done using the same APIs.

### 2.4 Generation of LLM outputs

LLMs evaluated the 24 papers separately, receiving the full extraction template and one of the 24 research papers in each API call. Text outputs were converted to JSON for analysis. The extraction template instructed models to only answer the extraction template if the paper was relevant to the research question (based on provided relevancy criteria). In cases where LLMs did not classify the paper as relevant, it was rerun with a modified prompt excluding this line to enable assessment of the LLM’s answers to the remaining questions.

### 2.5 Assessing LLM outputs

**Figure 2:**
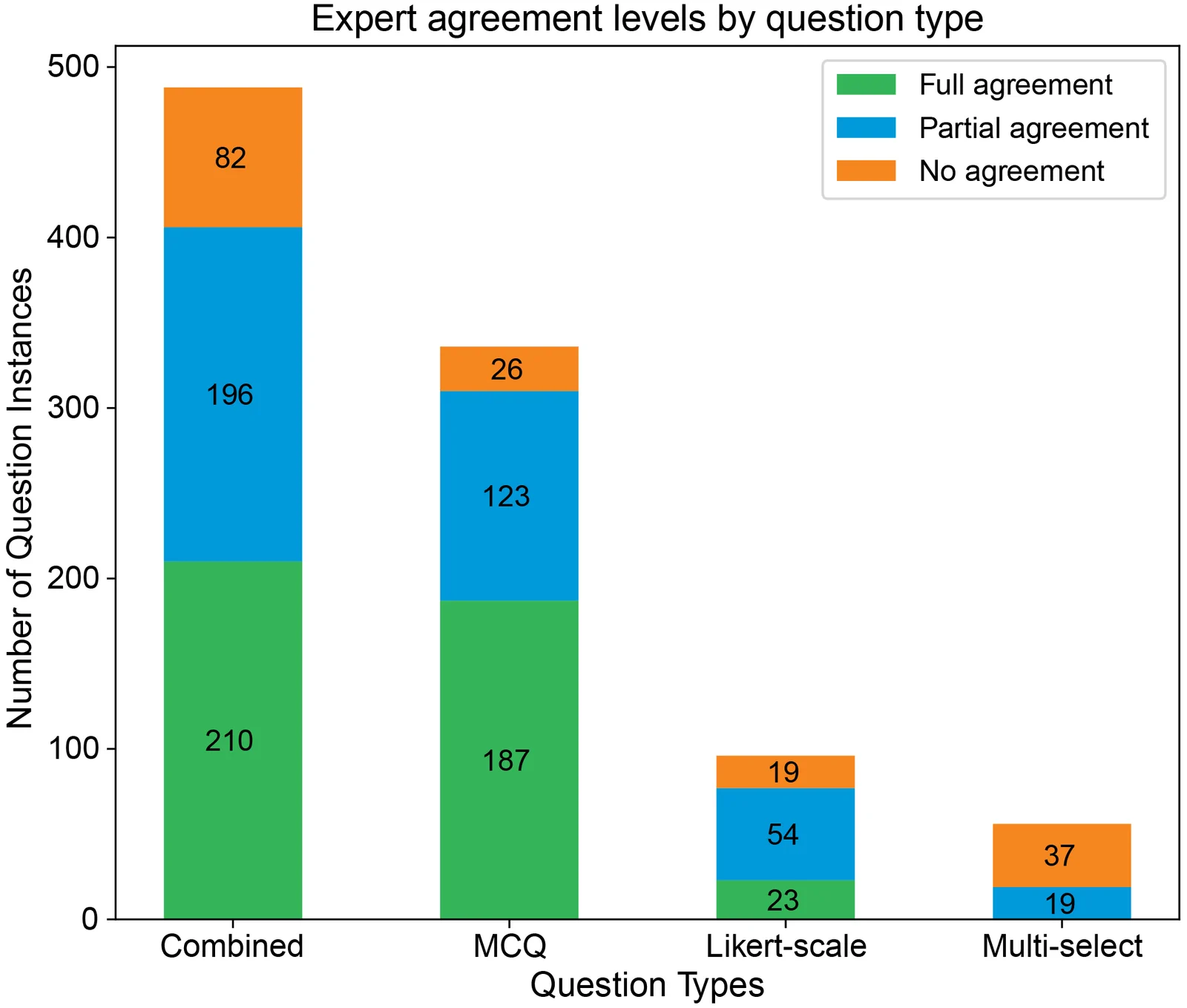
Expert agreement levels for different question types. Expert consensus (consisting of full and partial agreement) was reached in the majority of MCQ and Likert-scale question instances, while most multi-select question instances had no agreement. Overall, experts had full or partial agreement for the majority (406/488, 83.2%) of question instances (see “Combined”). The frequency of question instances where all three expert responses matched (“Full agreement”), where two expert responses matched (“Partial agreement”), and where no expert responses matched (“No agreement”), were determined for each question type.

Krippendorff’s Alpha was initially used to measure inter-rater agreement, however, since this was calculated across three (inter-expert) or four (expert-LLM) raters per question instance, differences in a single response had a large impact on agreement, but did not reflect the broader trends in the data. For example, despite experts reaching consensus (either two or all three experts agreeing) in 92.26% (310/336) of MCQs (**Figure 2**), Krippendorff’s Alpha for MCQ questions was 0.34, indicating poor expert agreement (Marzi et al., 2024). Therefore, question-specific metrics were designed. For clarity, question instances refer to individual questions and their responses (e.g., question 19 for paper one), while question items refer to the question as found in the extraction template (e.g., question 19 across all papers).

To determine whether LLMs performed at an expert level, their impact on the inter-expert agreement score distribution was assessed. If LLMs responded to the extraction template at the level of domain experts (i.e., provided responses similar to that of experts), then integrating their responses into the inter-expert group produced a distribution that was either indistinguishable from or higher than the inter-expert distribution. If LLM responses did not resemble that of experts (i.e., behaved as an outlier), then integration of their responses would produce a distribution significantly lower than that of the inter-expert agreement distribution. LLM rater groups were created by including the specified LLM’s response with the expert responses, and are referred to by LLM name or collectively as the expert-LLM groups.

#### 2.5.1 Multiple-choice questions

For each MCQ, all inter-expert, expert-LLM, and inter-LLM response pairs were compared, producing a score of one if choices matched and zero if they did not. These scores were then averaged to produce a normalized score per question instance for each rater group, referred to as the *MCQ match proportion*. Additionally, frequency of question instances with full expert agreement (three matching responses), partial agreement (two matching responses), and no agreement (no matching responses) was determined.

#### 2.5.2 Likert-scale questions

Considering response pairs could differ by a single rating level and still have good agreement; match proportions were deemed inappropriate. Likert-scale questions were therefore assessed in two ways:

1. To determine the distance between response values, for each question instance the absolute differences of response pairs (e.g., |*LLM_x_Score*−*Expert_y_Score*|) were averaged for each rater group to produce a normalized difference, referred to as the *scale distance*.
2. The scale distance is interpreted differently from other question type metrics, with lower values indicating greater agreement. Therefore to determine cumulative performance across all question instances from all question types, scale distances were inverted to enable assimilation (e.g., if the scale question was out of 5, and the scale distance was 1, the scale score would be 5 - 1 = 4). This is referred to as the *inverted distance*.

Considering the four Likert-scale question items were high impact (providing scores for paper reliability, strength of evidence, cumulative oncogenesis causal criteria score, and the impact of the paper on the MCP) sub-analysis was done for each question item.

#### 2.5.3 Multi-select questions

All multi-select questions were in the *“Microbial Oncogenesis Criteria”* template section, providing potential explanations (e.g., “Insufficient power”, “Concerns regarding paper reliability”, and “Appropriate methodology/tools”) for why a specific microbial oncogenesis criterion was supported, refuted, or remained uncertain. However, preliminary assessment of expert match rates revealed systematic expert disagreement (**Figure 2**), with experts selecting opposing options (e.g., “Appropriate methodology/tools” and “Inappropriate methodology/tools”). LLMs often returned either all provided options without indicating selection (Gemini Flash 2.5 and GPT-5-Nano), or provided a written explanation for each option. Therefore, no further analysis was performed, as these findings indicate confusion surrounding the question type (for both experts and LLMs) that precludes LLM assessment.

#### 2.5.4 Long-answer questions

Only question instances with three valid expert responses were used, with 75 question instances across eight question items assessed quantitatively and qualitatively (**Table 2**).

**Table 2:** Long-answer question items included in LLM assessment and their question numbers.

Long-answer question items with their corresponding question numbers	
Question number	Question
1.1*	Briefly explain why the paper is relevant, somewhat relevant, or irrelevant.
18	List the most important factor(s) in the methodology that influence(s) the strength of the study’s findings.
26.1**	If there are limitations, what are they?
28.1.4	State which kind of evidence is provided according to the tools to consider categories and identify the specific finding/s that fulfil or refute this criterion. (Epidemiologic Association)
28.2.4	State which kind of evidence is provided according to the tools to consider categories and identify the specific finding/s that fulfil or refute this criterion. (Histopathologic Association)
28.4.4	Identify the specific finding/s that fulfil or refute this criterion. (Experimental evidence of facilitation of oncogenesis)
28.5.4	State which kind of evidence is provided according to the tools to consider categories and identify the specific finding/s that fulfil or refute this criterion. (Molecular and Multi-omics evidence for interaction)
28.9.4	Identify the specific finding/s that fulfil or refute this criterion. (Plausibility)

* Q1.1 follows Q1, which includes the detailed classification criteria.

** Q26.1 follows Q26: “Are there any limitations to using this research as evidence for or against a plausible causative relationship between HMTV/MMTV-like virus and breast cancer?”.

##### 2.5.4.1 Quantitative analysis of long-answers

Long-answer responses were scored in two ways using an LLM judge (GPT-5, high-reasoning effort) to improve efficiency, supported by evidence that such models can approximate human judgment when assessing LLM outputs (Croxford et al., 2025; Reese et al., 2026). GPT-5 was selected due to its strong benchmark performance, and its scoring was validated on a subset of responses from each LLM to ensure reliability (see **Supplementary Tables 1**, **2**). In both scoring methods, the LLM judge was blinded to the model being assessed when LLM responses were included. The potential impact of self-preference bias (whereby LLM judges favor their own responses) was considered negligible, as the LLM judge was instructed to score the overlap of response pairs rather than identify the superior response. The scoring methods were as follows:

1. All response pairs were compared and their overlap scored on a continuous scale from 0–4 up to 1 decimal place (see **Supplementary Material** for scoring rubric, **Supplementary Figure 1**). These scores were averaged for each rater group to produce a normalized score for each question instance, referred to as the *pairwise overlap score*.
2. Each LLM’s response was compared to a clearly indicated reference answer (created by combining all three expert responses) and scored on a discrete scale from 0-4 (see **Supplementary Material** for scoring rubric, **Supplementary Figure 2**). This score is referred to as the *combined overlap score*.

##### 2.5.4.2 Qualitative analysis of long-answers

Manual evaluation was performed to classify gaps between LLM responses and the combined expert reference answer to distinguish between three possibilities: (1) LLM omissions, (2) LLM hallucinations and/or distortions, and (3) paper-supported additions by the LLM that were omitted from the reference answer. Omissions were defined as information included in the expert response(s) that was absent from the LLM response. Hallucinations referred to instances where LLMs provided information not resembling the original research paper (fabrications), while distortions referred to misinterpretations of the text. Paper-supported additions were defined as information included in the LLM response that was absent from the expert responses but supported by the original paper. Hallucinations, distortions, and paper-supported additions were distinguished by confirming whether the additional information was present in the original research paper.

### 2.6 Data analysis

Specialized metrics (described in Sec. 2.5) were used to determine inter-rater agreement. Data was dependent and determined to be non-normal using Shapiro-Wilk tests; hence findings were reported as median (IQR) when applicable, with the exception of aggregate performance, where findings were reported as mean ± SD. Per-paper scores were calculated by summing the agreement metric scores (MCQ match proportions, Likert-scale inverted distances, and long-answer pairwise overlap scores) per paper (isolated to question items with 3 expert responses). Aggregate performance was determined by averaging these per-paper scores. Friedman tests were used to determine whether there were differences in the distributions between groups (reported as test statistic *F_r_* and *p*-value), followed by *post-hoc* two-sided Wilcoxon Signed-Rank test with Bonferroni correction (reported as test statistic *W* and *p*-value) and calculation of the standardized effect size (*r*) when the group distributional difference (Friedman test) was determined to be significant (*p <* 0.05). Silverman’s rule was used to calculate bandwidth for kernel density estimation (KDE) plots, and distributions were extrapolated. A cross-classified mixed-effect model was fitted to determine whether LLMs tended to match specific expert response patterns, with normalized individual pairwise score as the outcome (score range of 0-1, individual pairs e.g., LLM_1_ to expert_1_, using MCQ match score, inverted Likert-scale distance, and long-answer pairwise overlap score), with LLM identity, expert identity, and their interaction as fixed effects, with question type as a covariate, and with question and paper numbers as random effects. All data was analyzed using Python 3.14.

## 3 Results

### 3.1 Inter-expert agreement

#### 3.1.1 MCQ agreement

Experts displayed strong agreement across MCQs (**Figure 2**), with full agreement in the majority of question instances (median MCQ match proportion of 1.00, IQR 0.33 - 1.00). No agreement (full expert disagreement) occurred in 7.7% (26/336) of MCQ instances and was confined to question items on applicability to African populations (5/336), sample size adequacy (6/336), and five microbial oncogenesis criteria (15/336). Most disagreements in the microbial oncogenesis criteria reflected differences in interpretation rather than opposing conclusions, while 1.2% (4/336) of instances involved directly contradictory assessments (e.g., one expert concluding that the findings supported microbial carcinogenicity and another concluding that they refuted it).

#### 3.1.2 Likert-scale agreement

**Figure 3:**
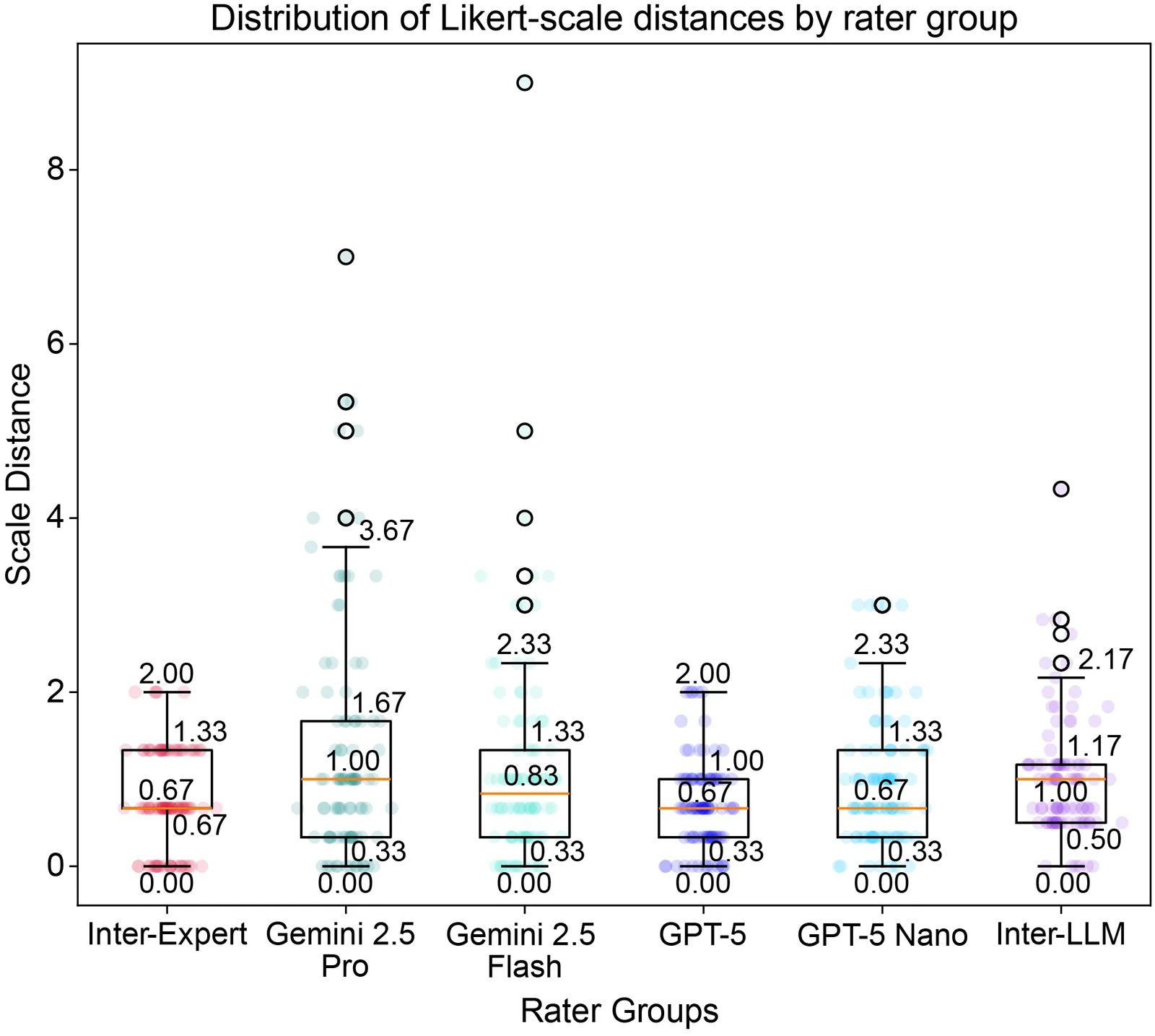
Distribution of Likert-scale distances across rater groups. Gemini 2.5 Pro’s Likert-scale ratings deviated substantially from experts, resulting in significantly increased scale distances when integrated, while Gemini 2.5 Flash, GPT-5, and GPT-5 Nano provided similar ratings to experts and did not alter the inter-expert scale distance distribution. Likert-scale distances were calculated per question instance (n=96) by averaging the differences between Likert-scale ratings for each rater pair (e.g., expert one and Gemini 2.5 Pro) in the rater group (e.g., Gemini 2.5 Pro). Outliers (circular outlines) were determined using Tukey’s method (values outside of interval *Q*_1_ − 1.5 × *IQR* and *Q*_3_+1.5 × *IQR*), whiskers indicate maximum and minimum values within interval. A Friedman test with *post-hoc* two-sided Wilcoxon-Signed-Rank tests were used to determine whether distributions differed.

Overall, expert Likert-scale ratings were closely clustered. For most question instances, experts had partial agreement with the outlier rating being one level away from consensus (**Figure 3**). Considering that individuals may differ in their interpretation and application of Likert-scale ratings, deviations of one level indicate good agreement around a central value. No agreement (full expert disagreement) occurred in 19.8% (19/96) of question instances and was most common for cumulative oncogenesis score (7/96) and paper reliability score (5/96). In most instances (15.6%, 15/96), expert responses spanned three consecutive Likert-scale levels (e.g., 1, 2, and 3), while 4.2% (4/96) of instances contained an outlier rating two levels removed from the nearest score.

Of the four Likert-scale question items, the strength of evidence and paper impact on MCP plausibility scores had the strongest expert agreement, followed by the paper reliability score, with score clustering and small scale distances. Cumulative oncogenesis score had the most disagreement with larger scale distances, reflecting moderate variability in interpretation and application of microbial oncogenesis criteria.

#### 3.1.3 Long-answer question agreement

**Figure 4:**
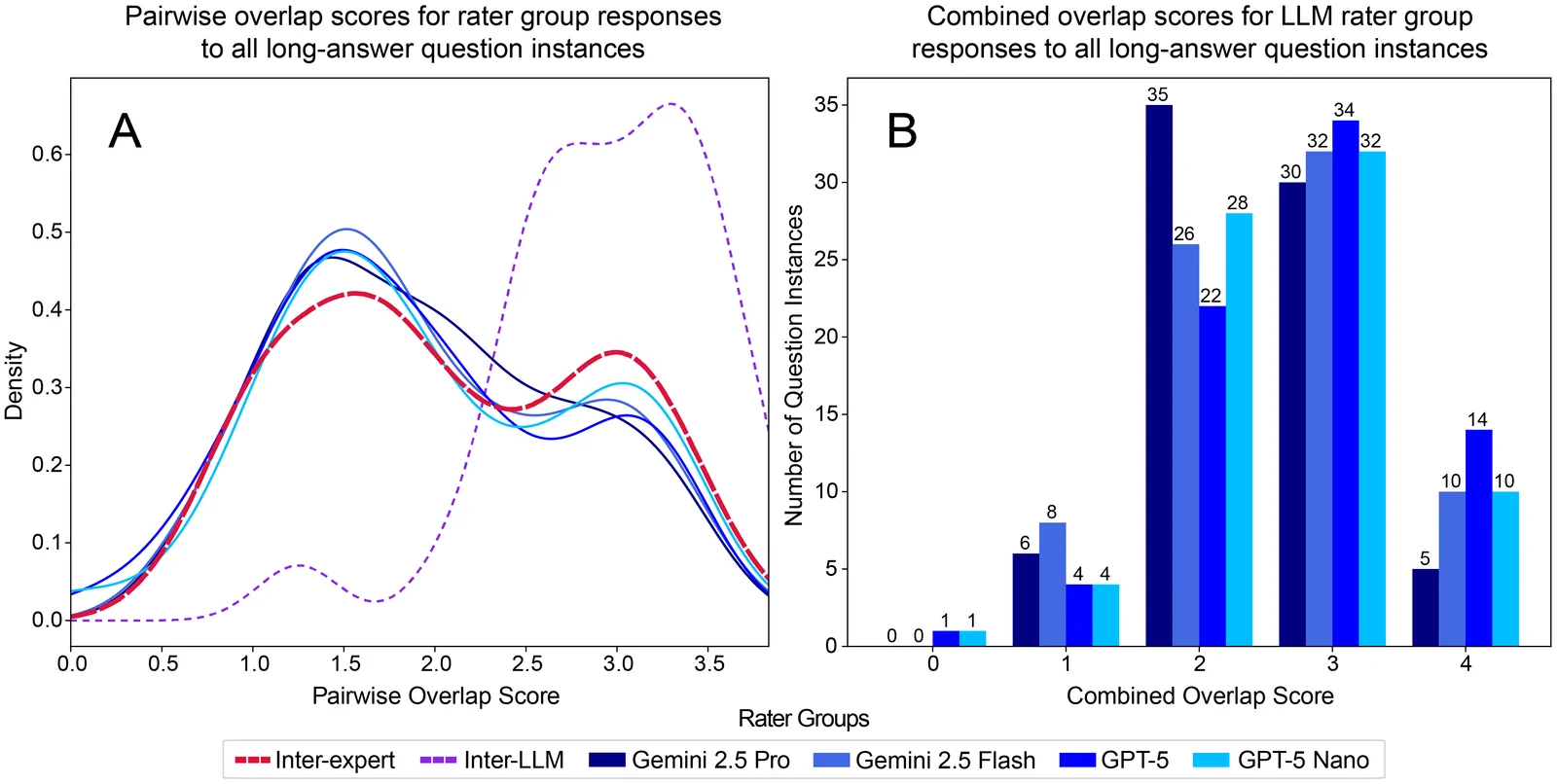
LLMs had higher combined overlap scores **(B)** than pairwise overlap scores **(A)**, indicating that LLMs tended to include information from each of the experts rather than a single expert. **(A)** LLM long-answer pairwise overlap score distributions were indistinguishable from the inter-expert distribution, indicating that LLM responses were as similar to individual experts as experts were to each other. Inter-LLM distribution differed significantly from inter-expert and expert-LLM distributions (all *p <* 0.0001*, r >* 0.75), indicating that LLM responses had greater overlap with each other than with experts, and had greater overlap than experts had with each other. Long-answer response pairs for 75 question instances were scored on their overlap by an LLM-judge (GPT-5, blinded to LLM being assessed) on a continuous rubric scale from 0-4. Scores for each question instance were averaged for each rater group to produce the pairwise overlap score. **(B)** LLM long-answer responses captured most key points found across three expert responses, with LLM responses predominantly scoring ≥2. A Friedman test found no significant differences in combined overlap scores across the different LLMs (*F_r_* = 6.48*, p* = 0.0906), indicating that response completeness did not differ by model size. For 75 question instances, LLM responses were compared to a reference answer created by combining three separate expert responses. Each LLM response was scored on whether it covered the key and/or minor points in the reference answer by an LLM-judge (GPT-5, blinded to LLM being assessed) using a discrete rubric scale from 0-4 (see **Supplementary Figure 2** for scoring rubric).

Expert responses captured similar key points, but differed on important nuances (**Figure 4A**). Pairwise overlap scores differed between question items. Q1.1 (see **Table 2** for full question items) had the highest scores, indicating strong agreement for reasoning on paper relevancy, while Q18 and Q26.1, which both focused on methodology, had the lowest pairwise overlap scores, indicating minor overlap with significant differences in responses. Manual evaluation found that experts included non-contradicting points focusing on different aspects of the paper, thereby producing a comprehensive combined response. Therefore, assessment of LLM responses using the combined overlap score method would mitigate these differences. Question items 28.1.4, 28.2.4, 28.4.4, 28.5.4, and 28.9.4, were combined for analysis due to their small sample size (six combined) and conceptual similarity (involved applying and extracting evidence for or against the microbial oncogenesis criteria). Manual evaluation of responses for these question items determined differences to be due to varying response foci and detail levels. Similarly to Q18 and Q26.1, this indicates that experts focused on different aspects of the question item and the paper’s findings, collectively producing a well-rounded response.

High levels of consensus and complementary responses among experts indicated that question items were clear and strengthened confidence in the human-validated test dataset as a means of benchmarking the LLMs.

### 3.2 Overall LLM performance across extraction template

#### 3.2.1 Aggregate performance

**Figure 5:**
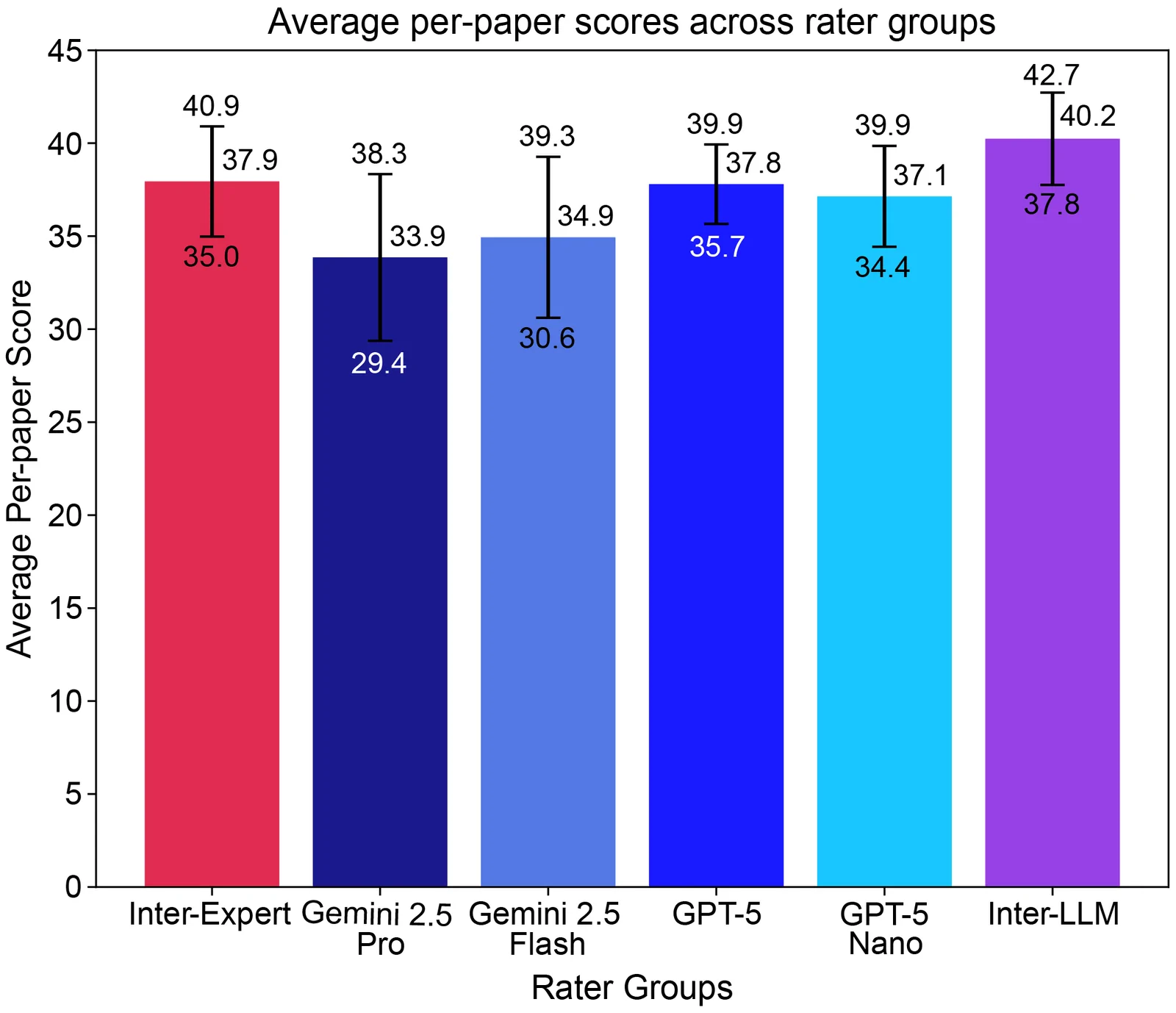
Average per-paper scores across rater groups. GPT-5 and GPT-5 Nano responses integrated seamlessly with expert responses across the 24 papers, producing no appreciable change in inter-expert agreement, while Gemini 2.5 Pro and Gemini 2.5 Flash’s responses were more inconsistent and tended to deviate from that of experts, significantly decreasing inter-expert agreement. Individual per-paper scores were determined by summing the MCQ match proportions, Likert-scale inverted distances, and long-answer pairwise overlap scores for each paper. Individual per-paper scores were averaged to produce the average per-paper score per rater group. Error bars represent ± *SD*.

Across the 24 papers, GPT-5 (*W* = 139.5*, p* = 1.0) and GPT-5 Nano (*W* = 115.0*, p* = 1.0) consistently gave similar responses to experts, producing no overall change in inter-expert agreement when integrated (i.e., when LLM responses were included as one of the human experts for analysis of agreement). In contrast, the responses of Gemini 2.5 Pro (*W* = 17.0*, p* = 0.0004*, r* = 0.78) and Gemini 2.5 Flash (*W* = 39.0*, p* = 0.0127*, r* = 0.65) tended to deviate from experts, resulting in significantly decreased inter-expert agreement when integrated (**Figure 5**). Inter-LLM agreement was significantly higher than inter-expert agreement (*W* = 45.0*, p* = 0.0267*, r* = 0.61), suggesting that LLM responses were more similar to each other than to expert responses, and exhibited greater inter-response similarity than that observed among experts.

Overall, GPT-5 and GPT-5 Nano produced responses most similar to domain experts, resulting in indistinguishable score distributions, while Gemini 2.5 Pro and Gemini 2.5 Flash tended to produce responses that differed from domain experts, resulting in significantly lower score distributions.

#### 3.2.2 Performance by question type

##### 3.2.2.1 MCQ performance

**Figure 6:**
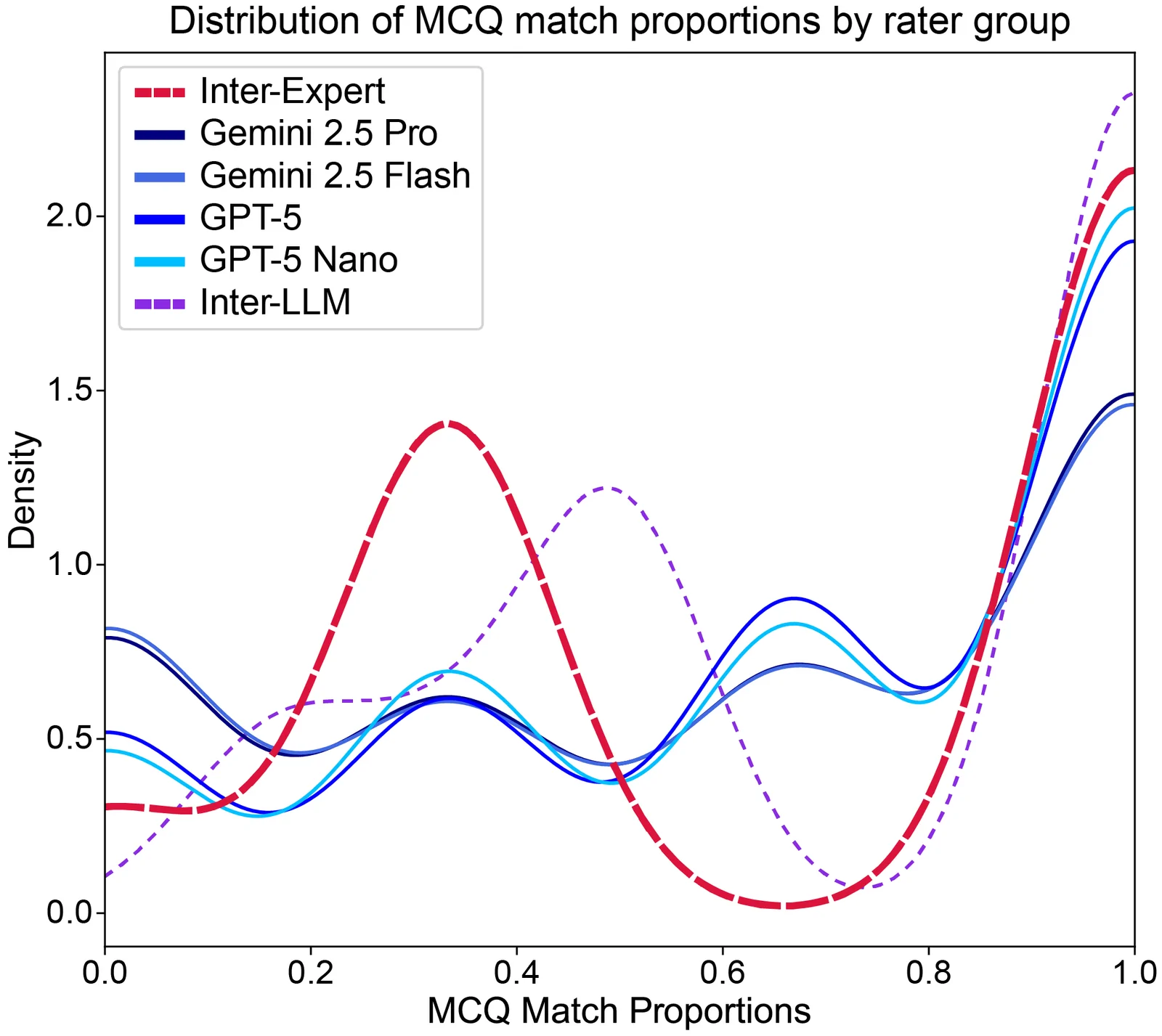
Distribution of MCQ match proportions across rater groups. All LLMs provided MCQ answers similar to experts, with integration of their responses resulting in either no change in inter-expert agreement (Gemini 2.5 Pro, Gemini 2.5 Flash), or a significant increase in inter-expert agreement (GPT-5, GPT-5 Nano). MCQ match proportions were calculated per question instance (n=336) by averaging the binary match rates for each rater pair (e.g., expert one to Gemini 2.5 Pro) in the rater group (e.g., Gemini 2.5 Pro rater group). Friedman test with *post-hoc* two-sided Wilcoxon-Signed-Rank tests were used to determine whether distributions differed.

All models provided similar MCQ answers to experts. GPT-5 (*W* = 3345.0, *p* = 0.0106, *r* = 0.76) and GPT-5 Nano’s (*W* = 2601.5*, p* = 0.0005*, r* = 0.79) MCQ answers were consistently similar to experts, tending to agree with expert consensus (either partial or full), therefore significantly increasing expert agreement when integrated. Gemini 2.5 Pro (*W* = 6110.0*, p* = 1.0) and Gemini 2.5 Flash (*W* = 6007.0*, p* = 1.0) tended to agree with the expert outlier, with integration of their MCQ answers having no effect on expert agreement (**Figure 6**).

##### 3.2.2.2 Likert-scale performance

Across the four Likert-scale questions, GPT-5 (*W* = 929.0*, p* = 1.00) and GPT-5 Nano (*W* = 1127.5*, p* = 1.00) rated the papers most similarly to experts, with integration of their ratings producing no overall change in expert scale distances. Gemini 2.5 Pro gave considerably higher ratings than experts, resulting in significantly greater expert scale distances (*W* = 639.0*, p* = 0.0013*, r* = 0.63). Similarly, while integration of Gemini 2.5 Flash’s ratings did not significantly alter expert scale distances (*W* = 769.0*, p* = 0.1981), these ratings were higher than experts’, resulting in larger scale distances (**Figure 3**). For all question items, higher ratings equated to greater leniency (for example, greater paper reliability or strength of evidence), indicating a consistent positivity bias in Gemini 2.5 Pro’s evaluations, with Gemini 2.5 Flash behaving similarly. Gemini 2.5 Pro and Gemini 2.5 Flash tended to agree with the expert outlier, while GPT-5 and GPT-5 Nano agreed with the expert consensus and outlier with similar frequency.

Overall, GPT-5, GPT-5 Nano, and Gemini 2.5 Flash rated papers similarly to experts across the different Likert-scale question items, although Gemini 2.5 Flash had a tendency to rate papers slightly more positively than experts, while Gemini 2.5 Pro consistently rated papers more favorably than experts, indicating a positivity bias in its evaluations.

##### 3.2.2.3 Long-answer performance

###### 3.2.2.3.1 Quantitative performance

Pairwise Overlap: LLM responses were as similar to individual expert responses as individual experts were to each other, with indistinguishable pairwise overlap score distributions between expert-LLM and inter-expert rater groups (**Figure 4A**, see **Supplementary Figure 1** for scoring rubric). LLMs gave very similar responses to one another, with significantly greater overlap between their responses compared to that of the expert-LLM and inter-expert groups (all pairwise tests *p <* 0.0001*, r >* 0.75).

Combined Expert Response Overlap: LLMs produced comprehensive responses that tended to capture key ideas dispersed across the three expert responses rather than aligning with a single expert, achieving greater combined overlap scores than pairwise overlap scores (**Figure 4B**, see **Supplementary Figure 2** for scoring rubric). Response completeness did not differ by model complexity (*F_r_* = 6.48*, p* = 0.0906), with smaller models (Gemini 2.5 Flash, GPT-5 Nano) capturing the same amount of key points as larger models (Gemini 2.5 Pro, GPT-5) across all question instances.

Overall, LLM long-answer responses were comprehensive, capturing key ideas from across the three expert responses. LLM responses were very similar, capturing the same amount of key points regardless of model size.

###### 3.2.2.3.2 Qualitative analysis

Omissions: Gemini 2.5 Flash and GPT-5 Nano had slightly less omissions than frontier LLMs Gemini 2.5 Pro and GPT-5 (**Table 3**). Per-question item analysis found that omissions for relevancy explanations (Q1.1) and responses involving extraction of findings supporting or refuting the microbial oncogenesis criteria (28.1.4, 28.2.4, 28.4.4, 28.5.4, 28.9.4) were generally minor and reflected differences in response focus between experts and LLMs, with singular exceptions for GPT-5 and GPT-5 Nano for the microbial oncogenesis criteria, while omissions for methodology-related question items (Q18 and Q26.1) were more significant.

**Table 3:** Of question instances where LLMs included information not in expert responses, almost all were paper-supported additions, with a few hallucinations and/or distortions found in smaller models (Gemini 2.5 Flash, GPT-5 Nano).

Frequency of omissions, hallucinations and/or distortions, and paper-supported additions in LLM long-answer responses				
LLM	Omissions^1^	Information not in expert responses	Hallucinations and/or distortions^2^	Paper-supported additions^3^
Gemini 2.5 Pro	68	51	0	51
Gemini 2.5 Flash	64	64	2	62
GPT-5	67	62	0	62
GPT-5 Nano	66	69	7	62

1 Omissions refer to information LLMs excluded from their responses that experts included in theirs.

2 Hallucinations and/or distortions refer to information included in the LLM response that was inaccurate to the original paper, either fabricated or representing a misinterpretation of the text.

3 Paper-supported additions refer to information included in the LLM response, absent from expert responses, but faithful to or found within the original paper.

While omissions were numerous, there were similar counts of paper-supported additions. Across 75 question instances, information gaps between experts and LLMs were manually assessed and categorized as either omissions, hallucinations and/or distortions, or paper-supported additions.

For methodology-related question items, LLMs tended to omit the same information. In some instances, experts included irrelevant information (for example, a description of results rather than methodological strengths or weaknesses), however, legitimate omissions included: (1) failure to comment on sample size or appropriateness, and (2) no mention of the country the study was conducted in, although models did occasionally mention the limited geographic generalizability of results. For Q26.1 specifically, LLMs were less likely to comment on potential errors or missing information in the papers than experts.

Out of all omissions, the most severe occurred in the *“Microbial Oncogenesis Criteria”* section for questions 28.2.4 (for *“Histopathologic Association”*) and 28.4.4 (for *“Experimental Evidence of Facilitation of Oncogenesis”*), by GPT-5 Nano and GPT-5 respectively. For both question instances, all three experts agreed that the paper helped fulfilled these criteria, while these LLMs did not. While both models’ reasoning were valid and indicated stricter adherence to the criterion’s definition than experts, GPT-5 Nano’s reasoning was inconsistent with its responses for this criterion in other papers, although these question instances were not included in the main analysis as they lacked three expert responses for comparison.

Overall, smaller models had less omissions than larger models, with Gemini 2.5 Flash having the least and Gemini 2.5 Pro having the most, although models tended to omit the same information. For most question items omissions were minor, with the exception of methodological questions where LLMs often failed to consider factors surrounding sample size, country of study origin, and potential errors in the papers.

Hallucinations and Distortions: Frontier models (Gemini 2.5 Pro, GPT-5) had no hallucinations or distortions across the 75 question instances, while smaller models (Gemini 2.5 Flash (64), GPT-5 Nano (69)) exhibited a limited number of such errors. Of the instances where Gemini 2.5 Flash (Singhal et al., 2023) and GPT-5 Nano (van de Schoot et al., 2021) introduced information not present in expert responses, 2 and 7 instances were classified as hallucinations and/or distortions (**Table 3**). These represented misinterpretations of nuanced, domain-specific methodological details rather than traditional fabrications, and occurred exclusively in methodology-focused questions (Q18 and Q26.1, see **Table 2** for full questions). None of the errors overlapped between the two models, indicating distinct failure modes.

*Gemini 2.5 Flash* Both errors were classified as distortions with minimal impact on evidence appraisal, and consisted of a misinterpretation of a PCR quality-control step and incomplete pooling of tissue sample type counts.

*GPT-5 Nano* GPT-5 Nano repeated errors across the two methodology-related question items for three papers, resulting in four unique hallucinations/distortions out of the seven instances. GPT-5 Nano appeared more prone to text misinterpretation than Gemini 2.5 Flash, with its errors impacting evidence strength appraisal. These included a misunderstanding of standard PCR terminology, a misreading of a 2×2 contingency table, conflation of metastatic status with overall cancer prevalence, and falsely stating that appropriate controls were absent, which GPT-5 Nano characterized as a critical methodological flaw weakening reliability.

Overall, frontier models Gemini 2.5 Pro and GPT-5 had no hallucinations and/or distortions in the 75 question instances assessed, while smaller models Gemini 2.5 Flash and GPT-5 Nano had minimal instances. Both smaller models’ errors represented misinterpretations of domain specific methodologies, however GPT-5 Nano’s had slightly more errors which were more severe, with downstream effects on the evaluation of the paper’s strength of evidence.

Paper-Supported Additions: LLMs tended to include the same paper-supported additions as one another, even for methodology-related question items (Q18 and Q26.1) requiring applied reasoning over extraction. The majority of question instances for these question items had paper-supported additions. For Q18, LLMs tended to extract more detailed methodological information (e.g., the exact controls and processes used), while for Q26.1, LLMs tended to include additional limitations which required integration of paper-based information and domain knowledge (e.g., acknowledging that FFPE tissues have inherent nucleic acid degradation risks that may impact viral detection). Although LLMs tended to include similar limitations to one another, often repeating the same (yet contextually appropriate) set of limitations, there were instances where the LLMs noted pivotal points that the experts missed.

### 3.3 Performance by template section

#### 3.3.1 Relevancy

Gemini 2.5 Pro was maximally sensitive, classifying all 24 papers as *Relevant*. Similarly, Gemini 2.5 Flash classified one paper as *Somewhat Relevant* (aligned with expert consensus) and GPT-5 classified two papers as *Somewhat Relevant*, one aligning with consensus and one where experts agreed the paper was *Irrelevant*. GPT-5 Nano demonstrated the strictest classification threshold, labeling 54.17% (13/24) of papers as *Somewhat Relevant*. In most cases, this aligned with at least a single expert; however, in one instance all experts classified the paper as *Relevant*, and in another all agreed it was *Irrelevant*.

LLM relevancy explanations consistently captured all major expert-identified points, with minor omissions (pairwise overlap scores ≥2 and combined overlap scores ≥3). Qualitative analysis of GPT-5 Nano’s responses found that it consistently identified relevant mechanistic or associative findings in the papers, explaining the high pairwise overlap scores, but concluded that absence of temporality or direct causal proof warranted a *Somewhat Relevant* classification.

Overall, LLMs demonstrated understanding of domain-specific nuances (both in the paper and in the classification criteria) and strong alignment with expert reasoning on paper relevancy.

#### 3.3.2 Paper integrity and reliability

##### 3.3.2.1 Identifying irrelevant references

Only GPT-5 claimed to find irrelevant references, and this was for two papers. Manual re-evaluation found GPT-5 to be incorrect for the first paper, although this seemingly had no downstream effect on GPT-5’s evidence appraisal. However, for the second paper, GPT-5 identified three references that did appear to be irrelevant to the sections they were cited in, which initial human evaluation missed.

##### 3.3.2.2 Identifying potential conflicts of interest

GPT-5 Nano claimed that university funding constituted “a potential COI concern in some assessments”, while Gemini 2.5 Flash claimed that the private laboratories the authors were affiliated with were contracted with the National Cancer Institute (NCI), which “could introduce an indirect, potential commercial interest”. In many instances, LLMs - most notably GPT-5 Nano - cited the paper’s own no conflict of interest declaration as reasoning for their response.

##### 3.3.2.3 Identifying contradictions

LLMs had difficulty consistently detecting human-identified contradictions in full-text papers, although these had minimal impact on the paper’s reliability. Gemini 2.5 Flash and GPT-5 were the only models to identify any human-identified contradictions, and this was for one of the 13 instances. Gemini 2.5 Pro and GPT-5 identified three and five contradictions, respectively, that the human evaluator missed. However, Gemini 2.5 Pro, GPT-5, and GPT-5 Nano had one, two, and four instances, respectively, where they either fabricated or misinterpreted a contradiction.

Gemini 2.5 Pro’s hallucination stated that values for a subgroup analysis did not add up to the correct value, yet in its response it demonstrated that they did, thereby fabricating a contradiction, while GPT-5 missed the authors’ justification for methodological decisions. GPT-5 Nano’s hallucinations were more severe, and included false inconsistencies in result values, sample sizes, and the counts in a 2 × 2 contingency table.

To determine whether failure arose from context window limitations or true inability, models were given the isolated sections of text, figures, and/or tables in which the human-identified contradictions were found. After repeated attempts, Gemini 2.5 Pro did not return any results, citing API rate limits despite request limit adherence. This isolation method improved performance considerably, with Gemini 2.5 Flash (11/13), GPT-5 (12/13), and GPT-5 Nano (9/13) detecting the majority of human-identified contradictions, indicating that prior failure was likely due to context window limitations rather than lack of ability. One of these contradictions required interpretation of an electrophoresis gel image, which Gemini 2.5 Flash and GPT-5 demonstrated, despite being generalist models without biomedical specialization. One contradiction, where the paper was inconsistent in which mouse models were used, was missed by all LLMs while the rest of the missed contractions varied between Gemini 2.5 Flash and GPT-5 Nano. In two of these isolated sections, Gemini 2.5 Flash and GPT-5 each identified two additional contradictions (a total of four each) that were missed by the human evaluator, with a total of six additional unique contradictions identified.

Overall, LLMs were largely unable to find the human-identified contradictions within the full-text papers, although Gemini 2.5 Flash and GPT-5 did identify contradictions the human evaluator missed. Gemini 2.5 Pro, GPT-5, and GPT-5 Nano all hallucinated some contradictions, although GPT-5 Nano’s were the only severe ones. Isolation experiments improved performance considerably, indicating that prior failure was due to context window limitations rather than inability or lack of domain knowledge.

##### 3.3.2.4 Paper reliability score

**Figure 7:**
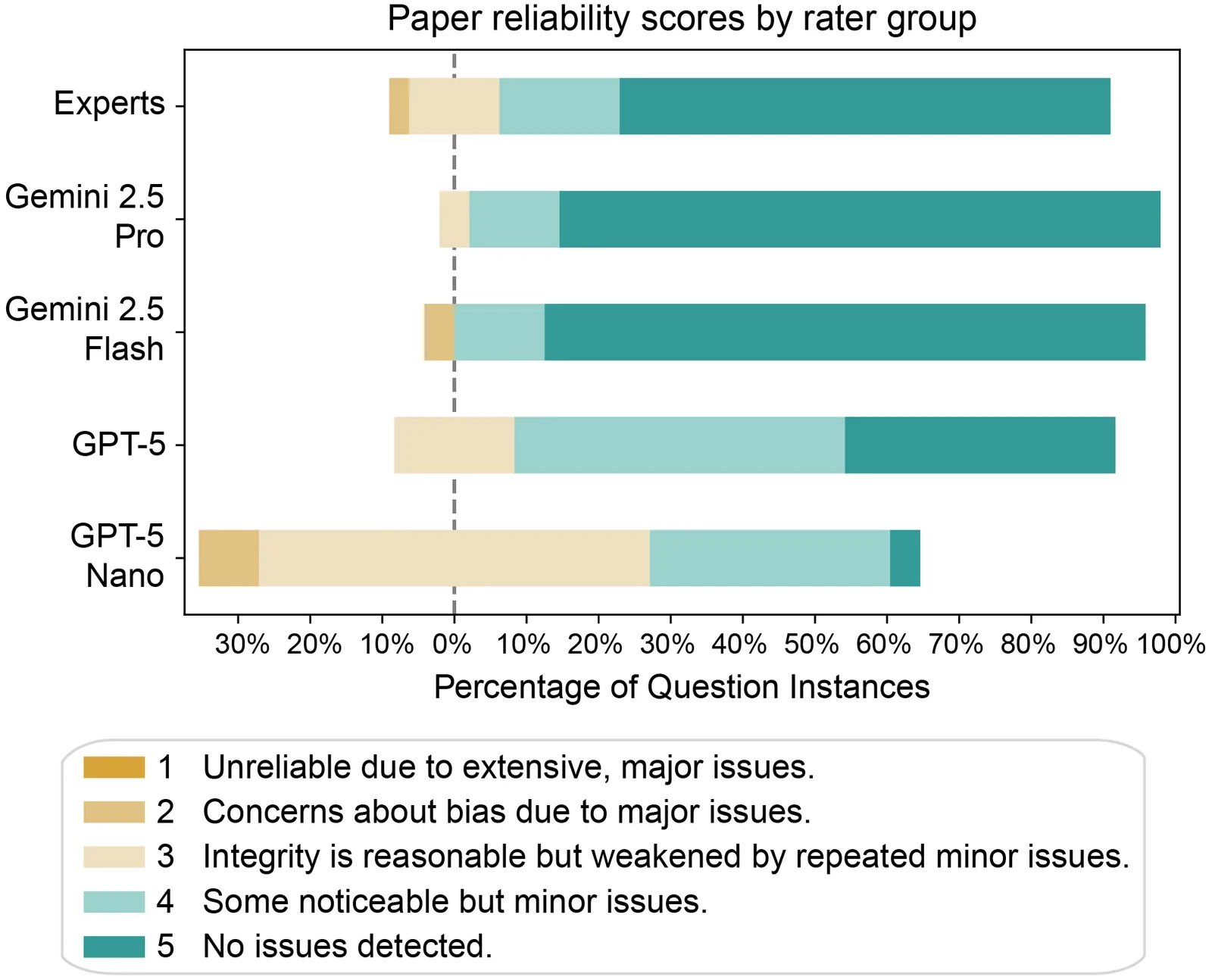
Distribution of paper reliability scores given by different rater groups (Likert-scale ratings, n=24). All three expert ratings for each question instance have been aggregated into the “Experts” group. Stacked bars represent the percentage of question instances assigned each score (1-5) by each rater group. Bars are aligned at 0% by the central rating value (3, dotted line), with lower scores (representing lower paper reliability) extending to the left, and higher scores (representing higher paper reliability) extending to the right. Gemini 2.5 Pro and Gemini 2.5 Flash gave very similar paper reliability scores to experts (predominantly 5s), while GPT-5 and GPT-5 Nano in particular gave lower paper reliability scores than experts (predominantly 3s and 4s), indicating more strict critique of paper reliability (although not significantly). Likert-scale rating meanings have been truncated for visualization (see **Supplementary Material** for extraction template with full meanings).

All models rated paper reliability similarly to experts, with no significant differences in the scale distances between the inter-expert and expert-LLM group pairs. Gemini 2.5 Pro and Gemini 2.5 Flash demonstrated the closest alignment with expert paper reliability ratings, followed by GPT-5, while GPT-5 Nano showed the greatest divergence, tending to rate paper reliability slightly lower than experts (**Figure 7**).

#### 3.3.3 Summary of paper contents

##### 3.3.3.1 Identifying important methodological factors influencing the strength of findings

**Figure 8:**
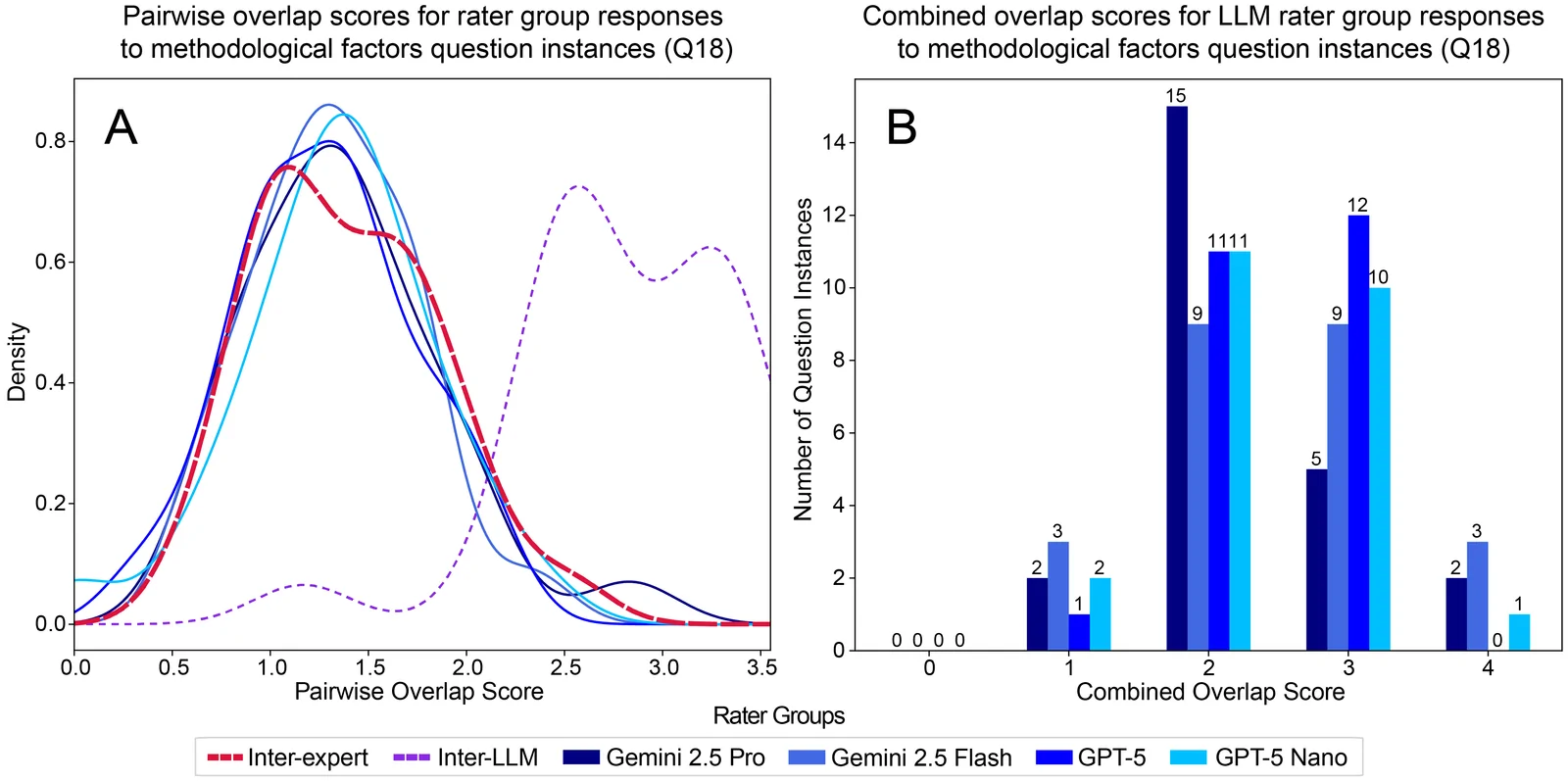
LLMs had higher combined overlap scores **(B)** than pairwise overlap scores **(A)** for Q18, indicating that LLMs tended to include important methodological factors from multiple experts rather than a single expert. Inter-LLM pairwise overlap scores were considerably greater than other rater groups **(A)**, indicating that LLMs often included the same methodological factors as one another in their responses. **(A)** LLM long-answer pairwise overlap score distributions were near indistinguishable from the inter-expert distribution. Long-answer response pairs for 75 question instances were scored on their overlap by an LLM judge (GPT-5, blinded to LLM being assessed) on a continuous rubric scale from 0-4 (see **Supplementary Figure 1** for scoring rubric). Scores for each question instance were averaged for each rater group to produce the pairwise overlap score. **(B)** LLM long-answer responses captured most key points found across three expert responses, with LLM responses predominantly scoring ≥2. For 75 question instances, LLM responses were compared to a reference answer created by combining three separate expert responses. Each LLM response was scored on whether it covered the key and/or minor points in the reference answer by an LLM-judge (GPT-5, blinded to LLM being assessed) using a discrete rubric scale from 0 to 4 (see **Supplementary Figure 2** for rubric).

Expert responses for this question item tended to include different, non-contradicting focus points, resulting in low pairwise overlap scores for inter-expert and expert-LLM groups (**Figure 8A**). However, LLMs tended to integrate key methodological considerations from multiple experts rather than aligning exclusively with a single expert, achieving higher combined overlap scores than pairwise overlap scores (**Figure 8B**). Gemini 2.5 Flash and GPT-5 demonstrated slightly better agreement with the combined reference response compared to Gemini 2.5 Pro and GPT-5 Nano, although distributions were broadly similar across models (**Figure 8B**). The majority of LLM responses had combined overlap scores ≥2, indicating capture of some key points with omissions (see Sec. 3.2.2.3.2). Gemini 2.5 Flash and GPT-5 Nano had hallucinations and/or distortions in some of their responses (see Sec. 3.2.2.3.2), indicating difficulty interpreting nuanced biomedical research methodology.

#### 3.3.4 Strength of evidence

##### 3.3.4.1 Identifying errors in statistical analysis or result presentation

GPT-5 and Gemini 2.5 Flash alone identified one of the four human-identified errors in result presentation (percentage miscalculation), and Gemini 2.5 Pro, GPT-5, and GPT-5 Nano identified two, three, and one errors, respectively, that were missed by the human evaluator. The human-identified errors the LLMs missed included two instances of incorrectly presented Venn diagrams (difference values did not exclude intersection values and intersection values were duplicated), and another instance where a results table had an incorrect category label. Additionally, Gemini 2.5 Pro and GPT-5 Nano both repeated hallucinated and/or distorted errors they gave in their contradiction analysis (see Sec. 3.3.2.3 for more details).

##### 3.3.4.2 Identifying limitations

**Figure 9:**
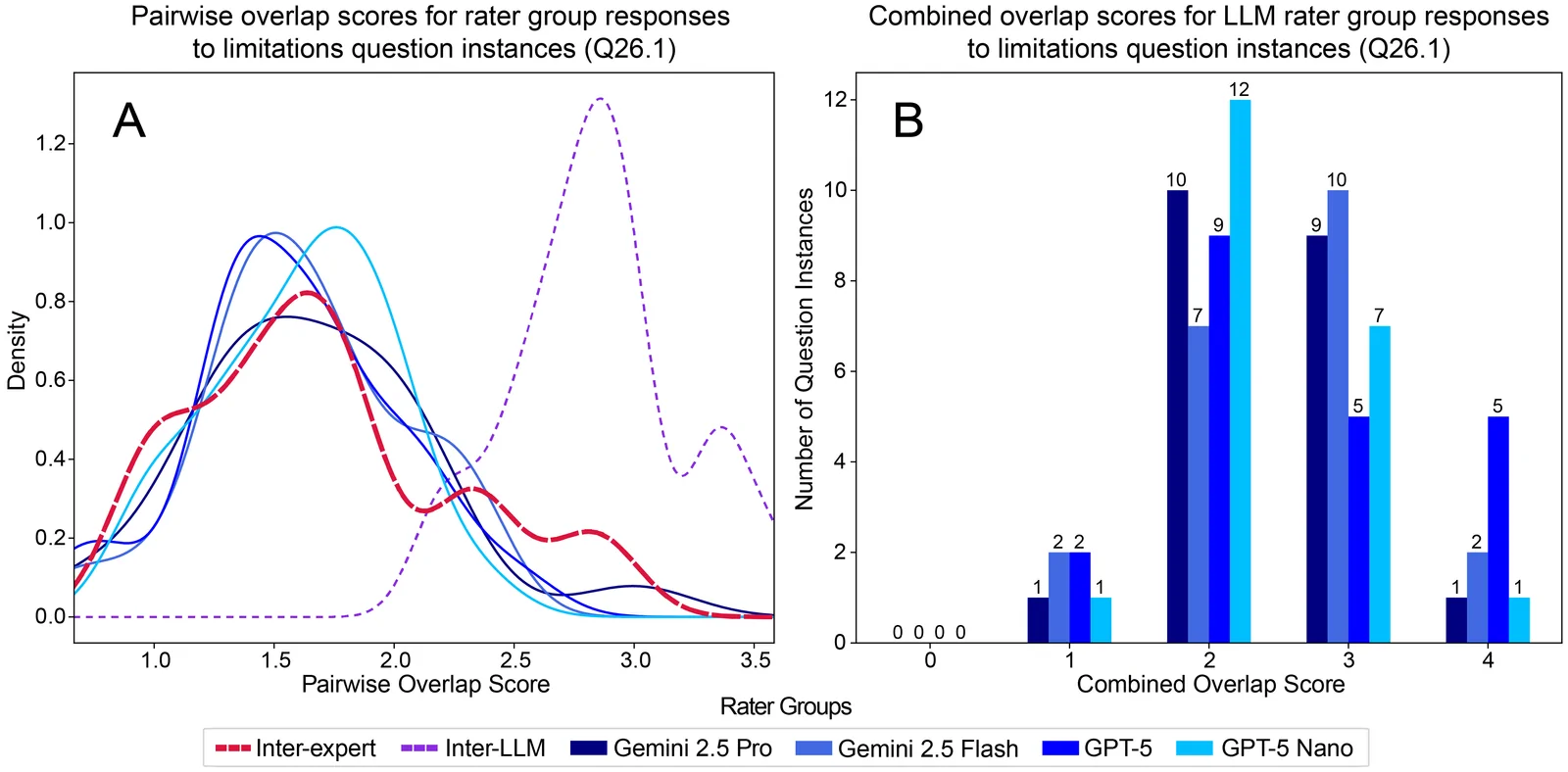
LLMs had higher combined overlap scores **(B)** than pairwise overlap scores **(A)** for Q26.1, indicating that LLMs tended to include limitations from multiple experts rather than a single expert. Higher inter-LLM pairwise overlap scores **(A)** indicated that LLMs included similar limitations to one another. **(A)** LLM long-answer pairwise overlap score distributions were similar to the inter-expert distribution. Long-answer response pairs for 75 question instances were scored on their overlap by an LLM-judge (GPT-5, blinded to LLM being assessed) on a continuous rubric scale from 0-4 (see **Supplementary Figure 1** for scoring rubric). Scores for each question instance were averaged for each rater group to produce the pairwise overlap score. **(B)** LLM long-answer responses captured most key points found across three expert responses, with LLM responses predominantly scoring ≥2. For 75 question instances, LLM responses were compared to a reference answer created by combining three separate expert responses. Each LLM response was scored on whether it covered the key and/or minor points in the reference answer by an LLM-judge (GPT-5, blinded to LLM being assessed) using a discrete rubric scale from 0 to 4 (see **Supplementary Figure 2** for scoring rubric).

LLMs tended to emphasize similar limitations to one another, while experts generally provided different (yet complementary) limitations, resulting in substantially higher inter-LLM pairwise overlap scores compared to the inter-expert and expert-LLM rater groups (**Figure 9A**). LLMs tended to integrate limitations from multiple experts rather than aligning with a single expert, with higher combined overlap scores than pairwise overlap scores for all LLMs (**Figure 9B**).

##### 3.3.4.3 Strength of evidence score

**Figure 10:**
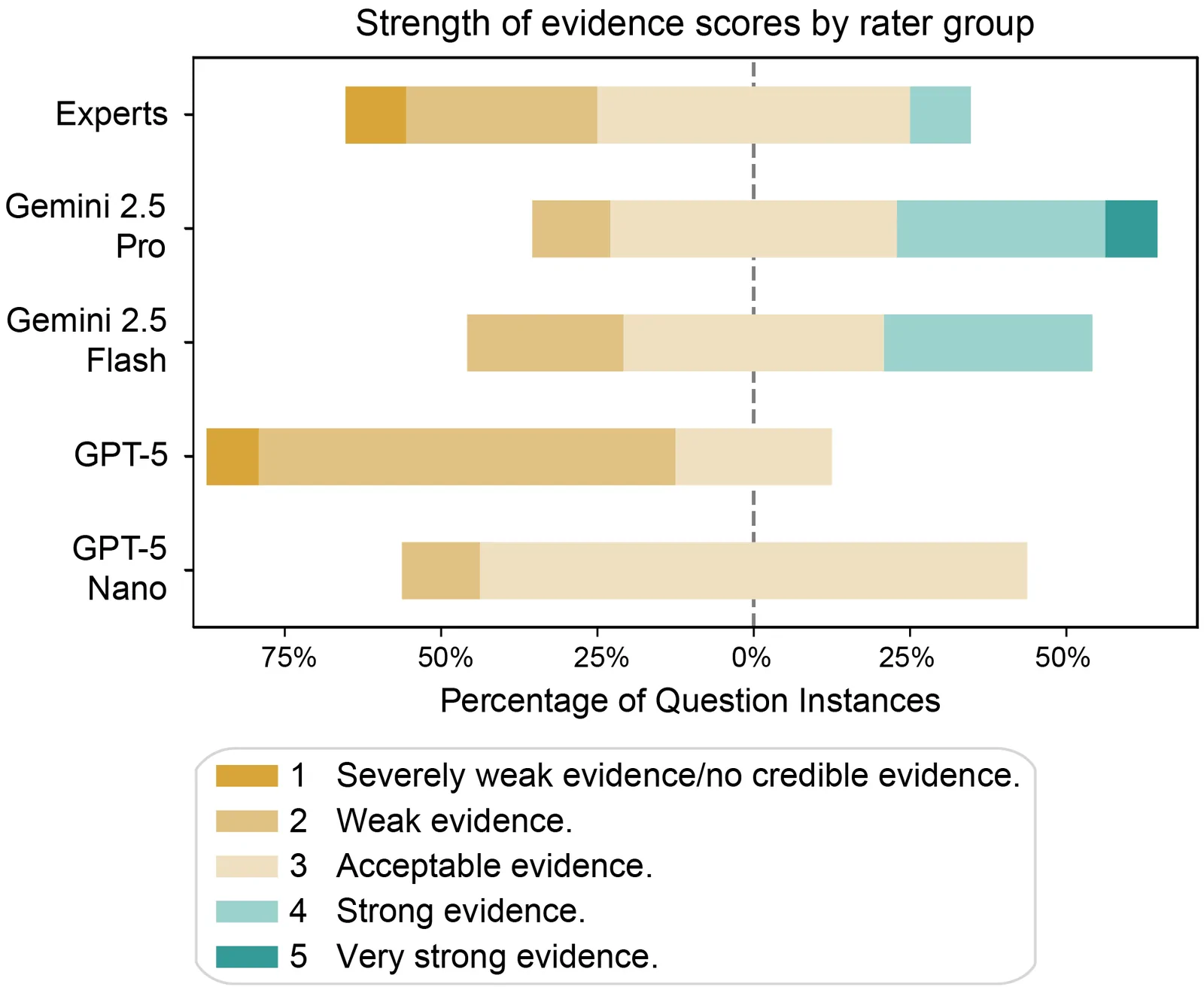
Distribution of strength of evidence scores given by different rater groups (Likert-scale ratings, n=24). All three expert ratings for each question instance have been aggregated into the “Experts” group. Stacked bars represent the percentage of question instances assigned each score (1-5) by each rater group. Bars are aligned at 0% by the central rating value (3, dotted line), with lower scores (representing lower strength of evidence) extending to the left, and higher scores (representing higher strength of evidence) extending to the right. Gemini 2.5 Pro and Gemini 2.5 Flash tended to rate strength of evidence slightly higher than experts (predominantly 3s and above), indicating greater leniency, while GPT-5 tended to rate strength of evidence slightly lower (predominantly 2s and below), indicating more strict evidence appraisal.

All models rated strength of evidence similarly to experts. Most LLM ratings were the same as or one level away from expert consensus (all LLM median scale distances ≤1), with no significant differences in the scale distances across the rater groups (*F_r_* = 13.42, *p* = 0.0197). When ratings did deviate from experts, Gemini 2.5 Pro and Gemini 2.5 Flash tended to rate strength of evidence slightly higher than experts, indicating greater leniency, while GPT-5 tended to rate strength of evidence slightly lower than experts, indicating a slightly stricter interpretation of evidence strength (**Figure 10**).

#### 3.3.5 Microbial oncogenesis criteria

##### 3.3.5.1 Identifying evidence type and findings supporting/refuting microbial oncogenesis criteria

Across the six questions analyzed, LLMs tended to include details from all three expert responses, with higher combined overlap scores than pairwise overlap scores. GPT-5 had the highest combined overlap scores (3.00, IQR 1.50 - 3.00), followed by Gemini 2.5 Pro (2.50, IQR 2.00 - 3.00) and Gemini 2.5 Flash (2.00, IQR 2.00 - 2.75), with GPT-5 Nano having the lowest scores (2.00, IQR 1.25 - 2.00), indicating that it tended to miss key points. Despite generally high performance, GPT-5, along with GPT-5 Nano, each had an instance where, contrary to expert consensus, they did not perceive a paper as helping to fulfill or refute the specified criterion, possibly indicating that these LLMs were occasionally more strict than experts in applying microbial oncogenesis criteria. Neither Gemini 2.5 Pro nor Gemini 2.5 Flash had this issue.

##### 3.3.5.2 Cumulative oncogenesis score

**Figure 11:**
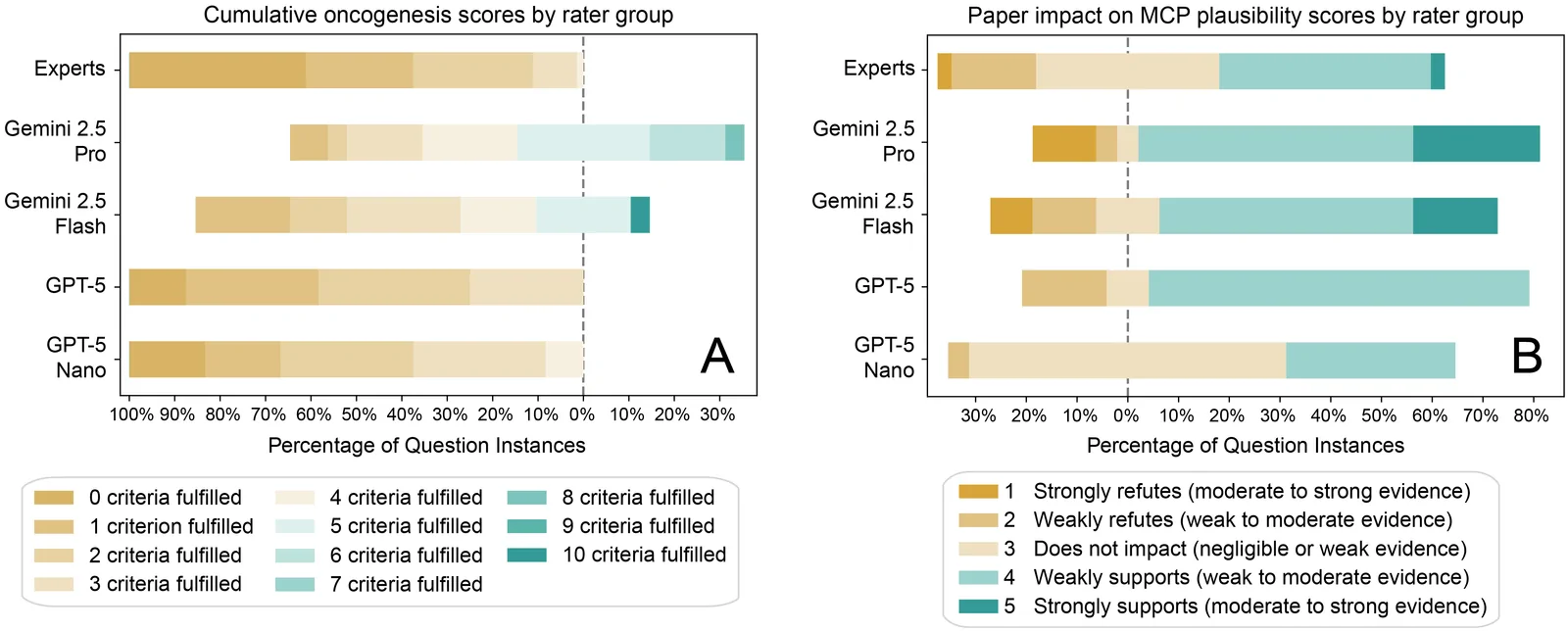
**(A)** Distribution of cumulative oncogenesis scores given by different raters (Likert-scale ratings, n=24). All three expert ratings for each question instance have been aggregated into the “Experts” group. Stacked bars represent the percentage of question instances assigned each score (0-10) by each rater group. Bars are aligned at 0% by the central rating value (5, dotted line), with lower scores extending to the left, and higher scores extending to the right. Cumulative oncogenesis scores were determined by summing scores for each of the 10 microbial oncogenesis criteria. Higher scores approaching 10 indicated fulfillment of more microbial oncogenesis criteria. Lower scores approaching 1 indicated either: (1) fulfillment of less criteria, or (2) fulfillment of some criteria combined with evidence to refute other criteria, as combinations of positive scores for certain criteria (paper helps support specific criterion) and negative scores for other criteria (paper helps refute specific criterion) may cancel out to produce a low positive score or a score of 0 depending on the combination. GPT-5 and GPT-5 Nano had cumulative oncogenesis scores most similar to experts (between 0 and 4), while Gemini 2.5 Pro and Gemini 2.5 Flash tended to state that papers supported more microbial oncogenesis criteria (in some instances ≥5 criteria for a single paper), and therefore gave higher cumulative oncogenesis scores than experts. **(B)** Comparison of paper impact on MCP plausibility scores (Likert-scale ratings, n=24) given by different raters. All three expert ratings for each question instance have been aggregated into the “Experts” group. Stacked bars represent the percentage of question instances assigned each score (1-5) by each rater group. Bars are aligned at 0% by the central rating value (3, dotted line), with lower scores (representing that the paper more strongly refuted the MCP’s plausibility) extending to the left, and higher scores (representing that the paper more strongly supported the MCP’s plausibility) extending to the right. Gemini 2.5 Pro, Gemini 2.5 Flash, and GPT-5 all tended to rate the paper’s impact on the MCP’s plausibility more highly, but Gemini 2.5 Pro and Gemini 2.5 Flash both gave more extreme ratings (1s for “Strongly refutes” and 5s for “Strongly supports”) compared to experts, GPT-5, and GPT-5 Nano.

GPT-5 (*W* = 86.0, *p* = 1.0) and GPT-5 Nano (*W* = 60.0, *p* = 1.0) applied microbial oncogenesis criteria in a manner similar to experts, with inclusion of their cumulative oncogenesis scores leaving inter-expert agreement unchanged. Gemini 2.5 Pro (*W* = 3.0, *p* = 0.0004, *r* = 0.86) and Gemini 2.5 Flash (*W* = 21.0, *p* = 0.0091, *r* = 0.75) were far more lenient than experts in stating a paper helped fulfill various microbial oncogenesis criteria, giving higher Likert-scale ratings and therefore significantly decreasing expert agreement (**Figure 11A**).

Gemini 2.5 Pro and Gemini 2.5 Flash had a tendency to state papers helped fulfill a given microbial oncogenicity criterion in contexts where evidentiary support was indirect or absent. For example, for a review of publicly available ecological data correlating mouse population changes with breast cancer incidence (Stewart and Chen, 2022), both Gemini models assigned cumulative oncogenesis scores of 8/10 and 10/10 respectively, indicating that they considered the paper to satisfy nearly all microbial oncogenesis criteria, including temporality, reproducibility/validation, dose-response relationship, molecular/multi-omics interaction, and even prevention. Notably, this behavior did not reflect misunderstanding of the papers, but rather an expansive interpretation of what constituted fulfillment of the oncogenesis criteria, with scoring justifications suggesting a susceptibility to ecological inference bias and false-cause reasoning.

Overall, GPT-5 and GPT-5 Nano applied the microbial oncogenesis criteria most similar to experts, while Gemini 2.5 Pro and Gemini 2.5 Flash deviated from experts, applying the criteria much more leniently.

##### 3.3.5.3 Rating impact of paper on MCP

LLMs rated each paper’s impact on the MCP’s plausibility similarly to experts, with no significant differences in the scale distances across the different rater groups (*F_r_* = 10.79, *p* = 0.0557), however, Gemini 2.5 Pro, Gemini 2.5 Flash, and GPT-5 all had a tendency towards rating the paper’s impact on the MCP’s plausibility higher, indicating slightly greater leniency in this regard than experts (**Figure 11B**).

### 3.4 LLM-expert similarity pattern analysis

A cross-classified mixed effects model investigating the pairwise scores of all question types (see Sec. 2.6) found no statistically significant evidence that any LLM preferentially matched specific experts (all *p >* 0*.*7), indicating that LLMs did not replicate the reasoning patterns of specific domain experts.

### 3.5 Stability of GPT-5 outputs

As GPT-5’s responses aligned most closely with experts, repeat experiments were performed to determine output stability. We ran 30 repeats on two papers, using a subset of six MCQs and two Likert-scale question items (see **Supplementary Material** Sec. 1.4 for included question items and the dataset for GPT-5’s outputs).

GPT-5 demonstrated high response stability across both papers. For the first paper, six of eight question items received identical responses across all runs, resulting in an overall stability of 92.5% (222/240 responses). For the second paper, five of eight question items received identical responses across all runs, resulting in an overall stability of 92.1% (221/240 responses). Across both papers, overall response stability was 92.3% (443/480 responses).

Where variation occurred, GPT-5’s responses generally remained closely aligned with expert assessments. For several MCQs, GPT-5 alternated between responses that matched either the expert consensus or the expert outlier. Similarly, variation in Likert-scale ratings was typically limited to a single rating level and remained close to expert evaluations.

Overall, despite occasional deviations, GPT-5’s responses were consistent across repeated runs and generally remained within the range of expert interpretations, indicating stable expert-aligned reasoning across repeated evaluations.

## 4 Discussion

**Figure 12:**
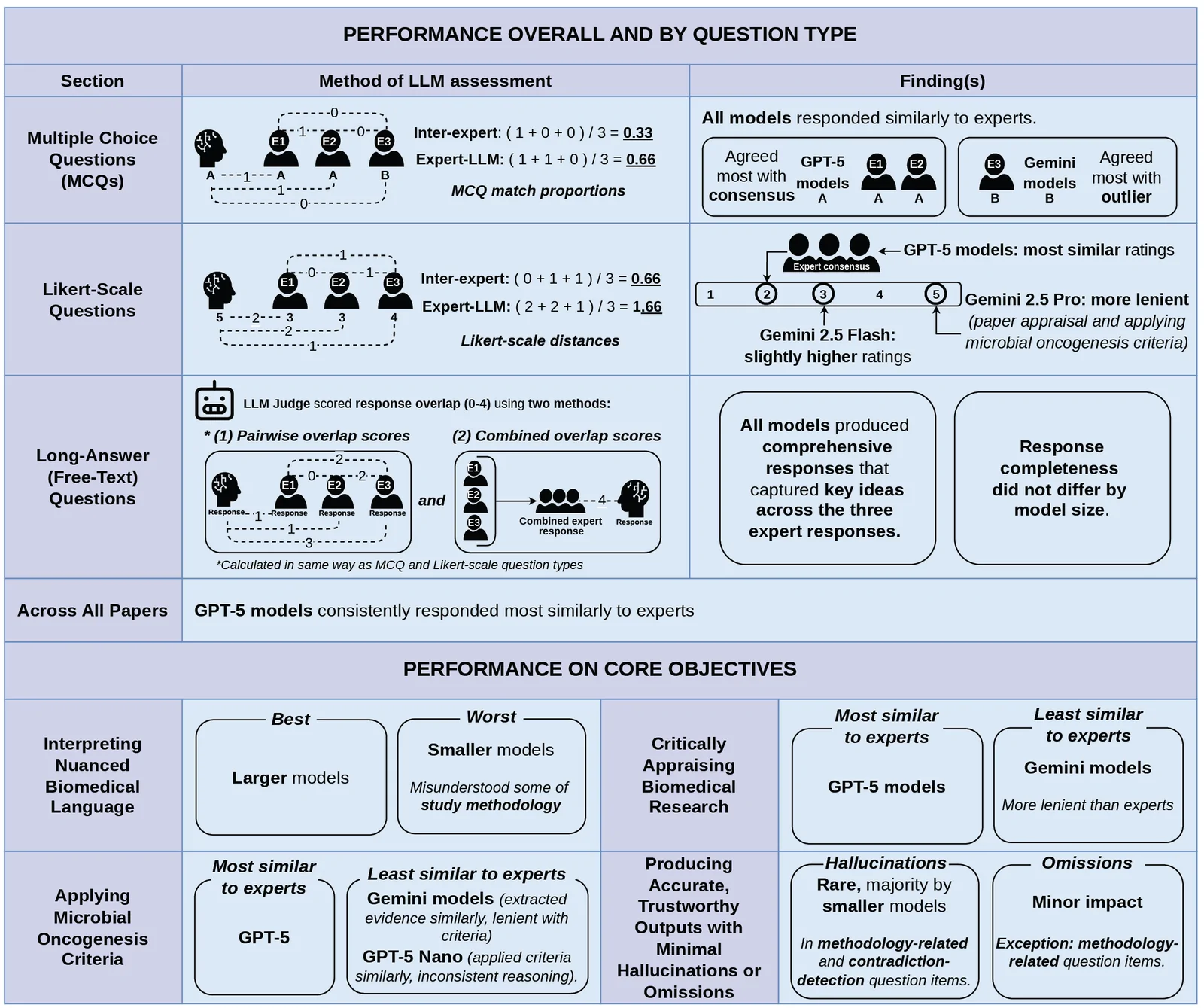
Summary of LLM performance by question type, overall, and on the core objectives. *“GPT-5 models”* refers to GPT-5 and GPT-5 Nano. *“Gemini models”* refers to Gemini 2.5 Pro and Gemini 2.5 Flash. *“Larger models”* refers to Gemini 2.5 Pro and GPT-5, while *“Smaller models”* refers to Gemini 2.5 Flash and GPT-5 Nano.

Our results show that pre-trained reasoning LLMs are capable of analyzing and evaluating individual biomedical research papers at the level of domain experts on most tasks when using a structured extraction and appraisal template, with minimal hallucinations (see **Figure 12** for summary of results).

The developed human-validated test dataset had strong inter-rater reliability, with high rates of full and partial agreement for MCQs (*>* 90%) and Likert-scale questions (*>* 80%, **Figure 2**), and close clustering of Likert-scale ratings even when consensus was absent (**Figure 3**). Long-answer responses demonstrated moderate overlap (**Figure 4A**), with variability primarily reflecting complementary reasoning rather than substantive disagreement between experts, as expected with nuanced domain-specific tasks. Considering this variability, we assessed whether inclusion of LLM responses altered the overall distribution of expert agreement (i.e., whether LLMs behaved as an additional expert), rather than requiring exact matches to expert responses. This may be a more effective strategy for evaluating LLMs in domains characterized by complex, nuanced tasks with non-contradicting inter-expert variability.

The exception to this pattern of strong inter-rater reliability was the multi-select (i.e., select all that apply) question type, which proved challenging for both experts and LLMs. Experts occasionally provided directly conflicting responses (e.g., one selecting “Appropriate methodology/tools” and another selecting “Inappropriate methodology/tools”), potentially reflecting differences in domain expertise and interpretation of study strengths and limitations. LLMs also struggled with this format, frequently selecting all available options or generating long-answer responses rather than selections, potentially reflecting poor exposure to this question type in their training data. These question items were excluded from further analysis as expert disagreement precluded the establishment of a reliable reference standard. The fact that both experts and LLMs encountered difficulties suggests that the issue lay primarily with the question type rather than either evaluator group. Future iterations of the extraction template will therefore replace multi-select items with a series of binary (yes/no) statements for each microbial oncogenesis criterion, accompanied by a justification field. Based on these findings, multi-select question formats are not recommended for either human or LLM-based evaluation of biomedical literature.

Across all 24 papers, GPT-5 and GPT-5 Nano performed most consistently and similarly to experts, while Gemini 2.5 Pro (*W* = 22.0, *p <* 0.0001, *r* = 0.75) and Gemini 2.5 Flash (*W* = 39.0, *p* = 0.0127, *r* = 0.65) behaved as outliers, significantly decreasing inter-expert agreement (**Figure 5**). However, performance varied across question types and items, with methodological appraisal questions and identification of contradictions being the most persistent areas of vulnerability.

Across MCQ items, LLMs performed similarly to experts - consistent with prior evidence that generalist LLMs (particularly GPT models) perform well on biomedical MCQ answering (Siam et al., 2025), with GPT-5 (*W* = 3345.0, *p* = 0.0106, *r* = 0.76) and GPT-5 Nano (*W* = 2601.5, *p* = 0.0005, *r* = 0.79) having significantly increased inter-expert agreement, agreeing most with expert consensus. Across Likert-scale questions, Gemini 2.5 Pro (*W* = 639.0, *p* = 0.0013, *r* = 0.63) had the greatest divergence from experts, significantly increasing scale distances by giving considerably higher Likert-scale ratings, indicating greater leniency in paper evaluation, while Gemini 2.5 Flash, GPT-5, and GPT-5 Nano gave similar scores to experts and therefore did not alter inter-expert distributions (**Figure 3**). When Gemini 2.5 Pro and Gemini 2.5 Flash’s ratings did match an expert’s, it tended to be the outlier, while GPT-5 and GPT-5 Nano matched the expert consensus and outlier with similar frequency. Repeat evaluations with GPT-5 (30 runs on two papers for a subset of eight question items) demonstrated response consistency across MCQ and Likert-scale question types (overall average response stability of 92.3%), with responses consistently matching or remaining closely clustered around expert ratings.

LLM long-answer responses were as similar to individual experts as experts were to each other, resulting in LLM long-answer pairwise overlap score distributions being indistinguishable from expert distributions (**Figure 4A**). Despite operating at a disadvantage against three domain experts, LLMs captured the majority of key points identified collectively by the expert group, demonstrating greater alignment with the combined expert reference answers than with individual expert responses (**Figure 4B**), suggesting integrative synthesis across perspectives and expertise. Despite differences in model size, complexity, and reported reasoning capacity, no significant superiority was observed across models in combined overlap scores, indicating functional convergence in synthesis capacity - however, limited instances of hallucinations and/or distortions by smaller models Gemini 2.5 Flash and GPT-5 Nano reduced their trustworthiness compared to frontier models Gemini 2.5 Pro and GPT-5 (see Sec. 3.2.2.3.2).

Interestingly, LLMs exhibited greater agreement with one another than with experts, and greater agreement than was observed between experts themselves. However, this effect was driven entirely by long-answer questions; agreement patterns for MCQ and Likert-scale items were comparable between LLMs and experts. Among the model pairings, Gemini 2.5 Pro and Gemini 2.5 Flash showed the greatest overlap in long-answer responses, followed by Gemini 2.5 Flash and GPT-5, and then GPT-5 and GPT-5 Nano. High inter-LLM agreement may partially reflect similarities in training data or learned representations, although the strong agreement observed between Gemini 2.5 Flash and GPT-5 suggests that shared LLM developer lineage alone cannot fully explain the findings. Another likely contributor is response length: LLMs typically provided substantially more detailed answers than experts, often listing multiple observations where experts highlighted only one or two key points. Consequently, LLM responses had a greater opportunity to overlap with one another, whereas agreement between experts and LLMs was inherently constrained by the concise nature of expert responses.

Given the inclusion of experts from multiple specialties, a cross-classified mixed-effects model was used to determine whether LLMs preferentially aligned with specific expert perspectives, thereby exhibiting specialty-specific reasoning patterns. However, there was no statistically significant evidence that any LLM consistently matched particular experts more closely than others (all *p >* 0*.*7). This finding suggests that the models did not replicate the reasoning patterns of individual domain specialists. One possible interpretation is that the LLMs integrated perspectives across multiple areas of expertise, producing responses that reflected a synthesis of expert viewpoints rather than alignment with any single specialist. This interpretation is consistent with the long-answer combined overlap scores, where LLMs achieved greater overlap with the combined expert responses than with any individual expert. An alternative explanation is that model responses were relatively random and thus did not systematically resemble any specific expert. However, the consistency of LLM performance across question types, together with their strong performance on long-answer questions, argues against largely random or inconsistent reasoning patterns and lends greater support to the integrative synthesis hypothesis. Nevertheless, the ability to detect expert-specific alignment may have been limited by the study design, as each paper was evaluated by only three experts and expert participation overlapped only partially across the dataset.

Building on the test dataset reliability and overall LLM performance assessment, we return to the core objectives: whether generalist, pre-trained LLMs can:

1. Interpret nuanced biomedical language,
2. Critically appraise biomedical research,
3. Apply microbial oncogenesis causal criteria to expert standards, and
4. Produce accurate, trustworthy outputs with minimal hallucinations or omissions

### 4.1 Interpreting nuanced biomedical language

While LLMs appeared to generally understand nuanced biomedical language, Gemini 2.5 Flash and GPT-5 Nano occasionally misinterpreted domain-specific methodological terminologies, leading to distortions and - in the case of GPT-5 Nano - downgrading of paper evidence (see Sec. 3.2.2.3.2). While smaller generalist models do not appear able to fully interpret nuanced biomedical language to the standard of domain experts, frontier models Gemini 2.5 Pro and GPT-5 performed sufficiently well, demonstrating understanding of complex domain-specific information, strengthening trust in their capabilities and enabling their use for systematic biomedical evidence synthesis processes such as ours.

### 4.2 Critically appraising biomedical research

Systematic differences in appraisal behavior were observed across models, suggesting architecture-dependent biases. Gemini 2.5 Pro significantly increased scale distances and demonstrated a consistent tendency toward more favorable appraisals of paper reliability (**Figure 7**), strength of evidence (**Figure 10**), and microbial oncogenesis plausibility (**Figure 11A**). Gemini 2.5 Flash behaved similarly, however, it did not produce significant changes in inter-expert agreement. In contrast, GPT-based models exhibited relatively more conservative scoring behavior that predominantly matched that of experts. These findings converge with our prior work demonstrating increased leniency in Gemini models when applying classification criteria (Dawood et al., 2025). While leniency increased sensitivity in the literature screening stage, it presently reduced alignment with expert judgment. In evidence synthesis contexts, such differences could meaningfully influence cumulative grading, prioritization, and downstream decision-making.

GPT-5 models demonstrated the highest alignment with expert critical appraisal. As all experts were recruited from a single institution, it is possible that some degree of shared institutional perspective influenced expert critical appraisal. However, several factors suggest that this is unlikely to explain the observed agreement. First, the expert panel was not educationally homogeneous, comprising individuals who received their undergraduate and postgraduate training from different institutions and who therefore entered the study with diverse academic backgrounds and methodological perspectives. Second, experts represented multiple specialties and departments, each of which place emphasis on different approaches to evidence evaluation, study design, and interpretation. This diversity was reflected in the fact that there was variance across the expert responses, and that they did not demonstrate complete consensus, with occasional disagreement in their assessments of paper reliability, strength of evidence, and microbial oncogenesis plausibility. Consequently, the expert evaluations cannot be viewed as the product of a single uniform institutional framework for critical appraisal. While it is conceivable that some shared perspectives could arise through a common institutional environment, given the vast and heterogeneous data sources used to train contemporary LLMs, it is unlikely that agreement with the expert panel resulted from exposure to appraisal patterns specific to a single South African institution. It is therefore more plausible that the observed alignment between GPT-5 models and experts reflects the models’ ability to identify broadly recognized indicators of paper reliability and strength of evidence than the replication of highly specific institutional appraisal norms.

While GPT-5 models appeared to display evidence appraisal capabilities comparable to experts, calibration or multi-model strategies may still be necessary when deploying LLMs in evaluative biomedical workflows requiring evidence appraisal. GPT-5 models remained more stringent in aspects of critical evaluation (tended to provide slightly lower ratings for paper reliability score and strength of evidence score than experts), while simultaneously failing to identify issues detected by human evaluators, including contradictions and methodological concerns. Thus, similarity in overall appraisal scores did not necessarily correspond to human-level identification of specific weaknesses within a study. Future studies could combine models with complementary strengths, for example use a model specifically fine-tuned for research quality assessment or trustworthiness evaluation to identify potential limitations, sources of bias, and methodological concerns, and then incorporate these findings into the appraisal process of a more generalist model such as GPT-5. Such architectures could potentially leverage the human-like scoring behavior observed in frontier LLMs while improving the depth and consistency of evidence appraisal, resulting in evaluations that more closely approximate expert review.

### 4.3 Applying microbial oncogenesis criteria

While Gemini 2.5 Pro, Gemini 2.5 Flash, and GPT-5 all demonstrated superior ability in extracting evidence to support and/or refute microbial oncogenesis criteria (adapted from (van Dorsten and Breiman, 2025)), GPT-5 applied and scored these criteria most similarly to experts, thereby producing cumulative oncogenesis scores comparable to experts (**Figure 11A**). GPT-5 had one instance of disagreement with expert consensus, but its reasoning was sound and indicated strict adherence to the provided microbial causal criteria definitions, increasing trust in its reasoning and consistency for future outputs. GPT-5 Nano appeared to apply and score the microbial oncogenesis criteria similarly to experts, yet had instances of inconsistent reasoning (see Sec. 3.2.2.3.2), in addition to less thorough evidence extraction than the other models (described in Sec. 3.2.2.3.2 and Sec. 4.4). Gemini 2.5 Pro and Gemini 2.5 Flash applied the criteria leniently, stating that criteria were fulfilled more often than experts did, thereby significantly decreasing inter-expert agreement on cumulative oncogenesis scores (**Figure 11A**). Further analysis found this leniency to be highly inappropriate, with susceptibility to ecological inference bias and false-cause reasoning, in addition to claims that indirect associative evidence (e.g., reviews of publicly accessible prevalence data) fulfilled multiple criteria requiring experimental or clinical evidence (e.g., temporality, dose-response relationship, and prevention).

Overall, Gemini 2.5 Pro, Gemini 2.5 Flash, and GPT-5 all demonstrated superior ability to extract evidence for nuanced microbial oncogenesis criteria to the level of domain experts, while GPT-5 alone applied the criteria most consistently and to expert standards. LLMs are therefore capable of applying and extracting evidence for nuanced criteria such as these, yet levels of competency and consistency in these tasks differ between models, requiring piloting and calibration of these tools before deployment. However, smaller generalist LLMs, such as GPT-5 Nano, may lack the domain knowledge and reasoning complexity to consistently apply and extract sufficient evidence for these criteria.

### 4.4 Producing accurate, trustworthy outputs with minimal hallucinations or omissions

Contrary to widespread concerns regarding prevalence of hallucinations in biomedical applications (Artsi et al., 2025; Kim et al., 2025), these were infrequent and largely restricted to smaller models, with most instances representing misinterpretations of the text (distortions) rather than typical fabrications (hallucinations). The majority of instances in which LLMs introduced information absent from expert responses represented paper-supported additions derived from the source paper rather than hallucinations or distortions (**Table 3**). LLMs more frequently had omissions in their responses, with most reflecting differences in emphasis or granularity rather than substantive misunderstanding. However, in two oncogenesis criteria question instances, GPT-5 and GPT-5 Nano failed to classify criteria as fulfilled where experts unanimously did so, suggesting potential rigidity in microbial oncogenicity criteria interpretation by these models (see Sec. 3.2.2.3.2). Conversely, LLMs frequently included paper-supported additions not identified by individual experts, suggesting differences in answer focus rather than inferential error. These low hallucination rates suggest that structured extraction templates, explicit criteria definitions, and constrained task framing may substantially mitigate hallucination risk. This is consistent with emerging evidence that structured prompting and reasoning scaffolds improve factual reliability (Anh-Hoang et al., 2025; Cheng et al., 2025), while other prompt refinements, including increased question specificity and minimum detail requirements, may reduce the frequency of omissions in future prompt iterations (Singhal et al., 2023; Kim et al., 2025).

Notably, hallucinations and distortions were isolated to two question groups: (1) methodology-focused questions, which required identification of study limitations and statistical interpretation, and (2) contradiction identification, which required understanding of nuanced biomedical language and interpretation of tables and figures.

Methodological critique demands integration of contextual domain knowledge (which generalist LLMs - especially smaller models - may lack), statistical reasoning, and inferential judgment, all of which are areas in which LLM limitations have been previously observed (Boye and Moell, 2025; Kim et al., 2025). Presently, when asked to identify potential limitations of studies, all LLMs tended to repeat the same set of limitations with minor changes to ensure contextual appropriateness, and were less likely than experts to comment on potential errors or information gaps. These failures illustrate the limits of domain-specific knowledge and skills in these generalist models, yet, there is clear potential to boost model performance in this regard, with instances where LLMs noted pivotal limitations that experts missed, and models tending to capture limitations from more than one expert in their responses. Overall, while integration of biomedical domain-specific models in a multi-model system may improve methodological appraisal ability, generalist model fine-tuning and prompt modifications may offer a similar performance boost while retaining the advanced reasoning abilities seen in these generalist models.

Poor performance in identifying contradictions within the papers was unsurprising, considering prior evidence that LLMs have difficulty identifying contradictions in provided full-text papers (Li et al., 2024) and isolated sentence pairs or text segments, with similar performance across models despite size differences in zero-shot settings (Tam et al., 2023). This difficulty is not unique to LLMs, with studies indicating that humans struggle to identify in-text contradictions, from peer-reviewers failing to identify inconsistencies between paper abstracts and full report results (Li et al., 2017), and studies demonstrating human difficulty in identifying contradictions in both full-text documents (Li et al., 2024) and isolated paragraphs, even when provided text segments are short (Otero and Kintsch, 1992). In contrast, when given the isolated paper sections containing the human-identified contradictions, LLMs performed considerably better, identifying close to all human-identified contradictions and detecting additional contradictions within these sections that the human evaluator missed. This indicates that while LLMs may have the ability to identify contradictions in research papers, current context window limitations likely preclude usability in this regard for full-text papers. This “context rot” poses an issue for our proposed pipeline, which relies on full-text papers. This requires the development of novel methods to circumvent large context size limitations and enable identification of errors for thorough evaluation of paper reliability and strength of evidence.

Overall, frontier LLMs’ outputs appear to be sufficiently accurate to be trusted in evidence synthesis workflows, demonstrating comprehensiveness with minimal hallucinations. Although omissions were frequent, their impact was predominantly minor, with LLMs still tending to include information spanning across the three experts responses rather than aligning with singular experts. Smaller LLMs’ outputs were less trustworthy, with higher (although still limited) instances of hallucinations and/or distortions, likely due to their poorer ability to interpret nuanced biomedical language compared to frontier LLMs. Methodology-related questions and contradiction detection remain the greatest areas of vulnerability for generalist LLMs due to domain knowledge and context window limitations.

### 4.5 Limitations

This study has several limitations. First, evaluation was restricted to a single MCP. This MCP was deliberately selected because of its unconfirmed status, conflicting evidence base, and predominance of associative rather than causal evidence, characteristics that are likely to resemble the intended real-world application of the system and many future MCPs of interest. Nevertheless, a single MCP cannot capture the full spectrum of evidence profiles encountered across potential MCPs. Although there is no obvious reason to expect the evaluated LLMs to be systematically biased towards or against this specific MCP, additional validation across MCPs with differing levels of evidential support would potentially strengthen confidence in the generalizability of these findings. In particular, future studies could evaluate MCPs approaching confirmed oncogenic status, including papers that challenge their plausibility, to assess whether model prior knowledge influences evaluation. Similarly, testing MCPs supported by sparse or emerging evidence could help determine whether model performance extends to more novel hypotheses. Consistent performance across such diverse MCPs could provide stronger evidence for the robustness and broader applicability of the proposed pipeline. Additionally, while the focus of this study is on applying this system to evaluating evidence on MCPs specifically, the choice of an MCP may limit generalizability of findings to other domains.

Second, the dataset comprised 24 papers, reflecting the intensive nature of the expert annotation process. While the extraction template consisted of 77 questions, allowing for extensive points of analysis across this dataset, 24 papers remain a small sample. Completion of the extraction template typically required more than two hours per paper, making large-scale expert evaluation impractical. Although this constrained the dataset size, it mirrors the real-world challenge that motivated the present work: the volume of biomedical literature far exceeds the capacity for comprehensive manual review. The ability of LLMs to perform comparable evaluations within minutes suggests a potential route to scaling such analyses, with experts serving in oversight and validation roles rather than conducting every assessment manually.

Third, the cohort of seven experts that provided responses for the human-validated test dataset were recruited from a single academic institution, and only three experts were assigned to each paper. While the study design of having independent review and extraction of information from papers was intentional to mimic current systematic evidence synthesis methods, a Delphi panel for consensus may have refined expert responses and further improved inter-expert agreement. Fourth, while the creation and use of a structured extraction template was intentional to standardize comparisons and enable control and transparency of LLM outputs, its use inevitably affects LLM reasoning patterns, which may improve model performance and reduce generalizability of findings to studies or domains that do not use such structured prompting.

Finally, LLM developers make frequent changes to their models, including updates to existing, already released models (e.g., updates to the Gemini 2.5 Pro model without access to previous versions), and further releases of brand new models (e.g., the release of GPT-5.1, a distinct model from GPT-5). Updates to existing models can result in these LLMs having different abilities and competencies than when they were originally tested, including potential decreases in performance. The models included in this study are considered stable due to shifted focus to and release of newer model ranges by Google and OpenAI (e.g., Gemini 3 and GPT-5.5 model ranges), which reduces concerns regarding changes in the included models’ performance in future. However, as newer models are released, the potential for older ones to eventually be depreciated (i.e., no longer accessible) remains. Furthermore, the included older models may fail to represent the full spectrum of these newer frontier LLMs’ abilities in the various discussed domains. However, our aim was to determine whether current LLMs were generally competent and trustworthy enough to be used for automated systematic evidence synthesis purposes, which we have found to be true. Each newly released LLM tends to beat prior models’ performance, and if this trend continues, the concerns found in the present study may no longer be applicable to these new models.

### 4.6 Conclusion

Overall, pre-trained reasoning LLMs (particularly GPT-5 models) were indistinguishable from experts across the structured biomedical evidence extraction and appraisal tasks. Hallucinations were rare in larger models (Gemini 2.5 Pro, GPT-5) under these constrained prompting conditions, but more frequent in smaller models (Gemini 2.5 Flash, GPT-5 Nano), although the rate of these errors could not be directly compared with human experts. These findings support integration of reasoning LLMs into AI evidence synthesis systems, particularly for evidence extraction and integrative summarization from individual biomedical research papers. However, methodological critique, scoring biases, and identification of contradictions remain key areas requiring further system refinement.

## Data Availability

The original contributions presented in the study are included in the article/**Supplementary Material**. Further inquiries can be directed to the corresponding author.
