# Correction: Artificial intelligence can match domain experts in evidence extraction and critical appraisal of microbial oncogenesis research publications

**DOI:** 10.3389/fcimb.2026.1964702

**Published:** 2026-08-18

**Authors:** Kaela Kokkas, Hairong Wang, Richard Klein, Nazir A. Ismail, Natalie Irwin, Mohammad Z. Moonsamy, Kubendran Naidoo, Jeremy Nel, Ekene E. Nweke, Raveen Parboosing, Emmanuel K. Sekyi, Rebecca T. van Dorsten, Bruce A. Bassett, Robert F. Breiman

**Affiliations:** 1 Department of Clinical Microbiology and Infectious Diseases, Faculty of Health Sciences, University of the Witwatersrand, Johannesburg, South Africa; 2 School of Computer Science and Applied Mathematics, University of the Witwatersrand, Johannesburg, South Africa; 3 Wits Machine Intelligence and Neural Discovery (MIND) Institute, University of the Witwatersrand, Johannesburg, South Africa; 4 Department of Clinical Microbiology and Infectious Diseases, National Health Laboratory Service and Faculty of Health Sciences, University of Witwatersrand, Johannesburg, South Africa; 5 Division of Medical Oncology, Department of Internal Medicine, University of the Witwatersrand, Johannesburg, South Africa; 6 Infectious Diseases and Oncology Research Institute (IDORI), Faculty of Health Sciences, University of the Witwatersrand, Johannesburg, South Africa; 7 South African Medical Research Council Vaccines and Infectious Diseases Analytics Research Unit, Faculty of Health Sciences, University of the Witwatersrand, Johannesburg, South Africa; 8 South African Medical Research Council Wits Antiviral Gene Therapy Research Unit, Faculty of Health Sciences, University of the Witwatersrand, Johannesburg, South Africa; 9 Department of Global Health, Rollins School of Public Health, Emory University, Atlanta, GA, United States; 10 National Health Laboratory Service, Johannesburg, South Africa; 11 Department of Molecular Medicine and Haematology, School of Pathology, University of the Witwatersrand, Johannesburg, South Africa; 12 Wits Research Institute for Malaria, Faculty of Health Sciences, National Health Laboratory Service, University of the Witwatersrand, Johannesburg, South Africa; 13 Division of Infectious Diseases, School of Clinical Medicine, Faculty of Health Sciences, University of the Witwatersrand, Johannesburg, South Africa; 14 Department of Surgery, Faculty of Health Sciences, University of Witwatersrand, Johannesburg, South Africa; 15 Division of Virology, University of Witwatersrand National Health Laboratory Service, Johannesburg, South Africa; 16 OncoVectra, London, United Kingdom

**Keywords:** large language models, artificial intelligence, critical appraisal, evidence extraction, evidence synthesis, evidence evaluation, biomedical literature, microbial oncogenesis

An incorrect **Funding** statement was provided. The correct funder for the article processing charges is the Infectious Diseases and Oncology Research Institute (IDORI). The correct **Funding** statement reads:

“The author(s) declared that financial support was received for this work and/or its publication. The work reported herein was made possible through funding by the South African Medical Research Council (SAMRC) through its Division of Research Capacity Development under the SAMRC Clinician Researcher Development Programme with funding received from the National Department of Health. Additional support was provided by the Wits Health Consortium and the Infectious Diseases and Oncology Research Institute (IDORI). Article processing charges for this publication were supported by IDORI.”

The original version of this article has been updated.

