# Supplementary material for: Artificial intelligence can match domain experts in evidence extraction and critical appraisal of microbial oncogenesis research publications
Source: Front Cell Infect Microbiol. 2026 Aug 6;16:1876326. doi: 10.3389/fcimb.2026.1876326 (PMC13531347; doi:10.3389/fcimb.2026.1876326)
Supplement: Supplementary file 1 [file DataSheet1.pdf]

Supplementary Material

1 SUPPLEMENTARY DATA

1.1 Figures

Figure S1: Assessment prompt for LLM judge (GPT-5, high reasoning effort) for pairwise long-answer comparisons (pairwise overlap scores).

You are serving as an impartial medical research evaluator. Your task is to score how well two responses match each other in meaning and nuance. The topic is whether HMTV / MMTV-like virus can cause breast cancer in humans.

SCORING RUBRIC:

- 0: No overlap: Responses share no common points.
- 1: Poor: Some minor overlap exists, but the responses differ substantially on key points or important nuances.
- 2: Fair: Several key points overlap, but significant differences remain in multiple important nuances.
- 3: Good: Most key points overlap, but the responses differ on one or two minor points.
- 4: Excellent: The responses are fully comprehensive, capturing all the same points and nuances.

DATA TO EVALUATE:
Question: "{question}"
{response 1}
{response 2}

INSTRUCTIONS:
Respond *only* with a JSON object containing:

1."score": A number from 0 to 4, expressed to one decimal place.
2."justification": A brief explanation for your score (2-4 sentences).

If one response includes information not in the other response, begin your justification with:
[EXTRA_INFO]
If this extra information is also irrelevant to the question, begin your justification with:
[EXTRA_INFO] [IRRELEVANT_INFO]
Do not include any text before or after the JSON.

Figure S2: Assessment prompt for LLM judge (GPT-5, high reasoning effort) for LLM to combined expert response comparison (combined overlap score).

You are a medical researcher assessing the ability of LLMs to evaluate and summarise biomedical research papers. An LLM has been given a research paper on the topic of whether HMTV / MMTV-like virus can cause breast cancer in humans, along with a set of questions to answer based on the paper. Score the LLM answer using the scoring rubric below based on its completeness, accuracy, succinctness, and focus compared to the three provided expert reference answers.

Scoring Rubric:

- 0: Irrelevant or Incorrect: The generated answer is completely off-topic or factually wrong compared to the references.
- 1: Poor: The answer includes a minor point but misses the main idea(s) or important nuances captured in the references.
- 2: Fair: The answer captures some of the key ideas but is incomplete, misses some nuances, includes irrelevant information, or is more verbose than necessary.
- 3: Good: The answer includes most of the key points from the references but either omits a minor point, includes some irrelevant information, or is slightly more verbose than necessary.
- 4: Excellent: The answer is comprehensive, succinct, and does not contain irrelevant information. It is as good as, or better than, the expert reference answers or combination thereof.

Here is the data to evaluate:
Generated Answer:
{llm_response}
{reference_block}

INSTRUCTIONS: Provide your evaluation in a JSON format with two keys:

1. "score": An integer from 0 to 4.
2. "justification": A brief explanation for your score. If the LLM answer includes any information not present in the references, begin your justification with the flag "[EXTRA_INFO]".

Do not add any text before or after the JSON object.

1.2 Tables

Table S1. Manual scoring of pairwise overlap score was done for two question instances (all LLMs included, resulting in 30 scores for comparison). The table shows the number of question instances where the LLM-judge score was an exact match to the human-judge score, and where the LLM-judge score was higher or lower than the human-judge score. The human and LLM-judge scores, along with their respective justifications, can be found in the dataset. Initial human scores were discussed by the research team. The majority of LLM-judge scores were very close to the human-judge scores ($\leq$1 point difference). In total, 10/30 scores were exact matches, 11/30 deviated by $\leq$0.5 points, 7/30 deviated by 1 point, and 2/30 deviated by 2 points.

**Comparison of human and LLM-judge pairwise overlap scores**			
**Evaluated group**	**Exact Match**	**Higher than human judge**	**Lower than human judge**
Experts	1	5*	0
Gemini 2.5 Pro	3	1**	2**
Gemini 2.5 Flash	3	1***	2**
GPT-5	3	2***	1***
GPT-5 Nano	0	5***	1***
Overall	10	18	6

* Three deviated by $\leq$0.5 points, while two deviated by 2 points.
** Deviated from human score by $\leq$0.5 points.
*** Deviated from human score by $\leq$1 point.

Table S2. Manual scoring of combined overlap score was done for eight question instances (two per LLM). The table shows the number of question instances where the LLM-judge score was an exact match to the human-judge score, and where the LLM-judge score was higher or lower than the human-judge score. The human and LLM-judge scores, along with their respective justifications, can be found in the dataset. Initial human scores were further discussed by the research team. The majority of LLM-judge scores matched the human-judge scores exactly (6/8), with 2/8 scores deviating by 1 point.

**Comparison of human and LLM-judge combined overlap scores**			
**Evaluated LLM**	**Exact Match**	**Higher than human judge**	**Lower than human judge**
Gemini 2.5 Pro	1	1*	0
Gemini 2.5 Flash	1	0	1*
GPT-5	2	0	0
GPT-5 Nano	2	0	0
Overall	6	1*	1*

* Deviated from human score by 1 point.

1.3 Extraction Template (Questionnaire)

Category one questions (answered by domain experts) are colored black, while category two questions (Q2-11.1, Q13-16, Q19-21, and Q23-25.1, answered by clinician-scientist trainee) are colored blue. Note that "SAQ"s in the template are referred to as MCQs in the manuscript, while "MCQ"s in the template are referred to as multi-select questions in the manuscript.

You are a medical researcher analysing the literature to evaluate the plausibility of HMTV/MMTV-like virus as a causative agent of breast cancer in humans. For each paper, you will generate a summary and evaluation of its findings by answering 77 predefined questions. These individual summaries will contribute to a final determination of whether HMTV/MMTV-like virus plays a causative role in human breast cancer and whether this warrants further research. To achieve this, carefully review the given paper and respond to questions 1 through 30, some of which are in SAQ or MCQ format, while others are long answer questions.

Relevancy

1. How relevant is this article in determining whether it is plausible that HMTV/MMTV-like virus can cause breast cancer in humans (relevant, somewhat relevant, or irrelevant)? A relevant article would fulfil either of the following criteria: 1) gives causal evidence for whether or not HMTV/MMTV-like virus or its species/family of microbes cause breast cancer or other cancers either in humans or animal models, or 2) contains a theoretical model for how HMTV/MMTV-like virus might cause breast cancer, irrespective of whether there is experimental evidence. A somewhat relevant article would fulfil either of the following criteria: 1) mentions both HMTV/MMTV-like virus and breast cancer, but does not give any causal arguments or evidence for HMTV/MMTV-like virus causing breast cancer, or 2) contains evidence examining whether other microbes cause breast cancer, if the findings can plausibly generalise to HMTV/MMTV-like virus and breast cancer. An irrelevant article would be a paper that does not fulfil the criteria for the relevant or somewhat relevant classifications.

a) Relevant
b) Somewhat relevant
c) Irrelevant

1.1 Briefly explain why the paper is relevant, somewhat relevant, or irrelevant.

Only continue to answer the questions after this if the paper is relevant.

Paper Bibliographic Details

2. Title of paper

3. Publication date

4. Journal of publication

5. Study design (e.g., randomised control trial, cohort study, case-control study, cross-sectional studies, laboratory study, etc) **6.** What country was the research conducted in?

7. DOI

Paper Integrity & Reliability

8. Are the references relevant to the topic of this paper and the sections of the text that they are cited in?

a) Yes, all references are relevant.
b) No, there were irrelevant references.

8.1 If any of the references were irrelevant, list them and state why they are irrelevant.

9. Was there any evidence of conflict of interest in this paper? This may include, but is not limited to, funding of research that could compromise the researchers' objectivity, and patent ownership related to the research.

a) Yes, there was evidence of a conflict of interest in the paper.
b) No, there was no evidence of a conflict of interest in the paper.

9.1 If there was evidence of a conflict of interest, what was it?

10. Are there any contradictions within the paper? Examples of contradictions include inconsistencies in reported sample sizes and participant characteristics, methodological discrepancies between stated procedures and actual implementation, or variations in how results are reported across different sections of the paper.

a) Yes, there were contradictions within the paper.
b) No, there were no contradictions found within the paper.

10.1 If there were contradictions, list them.

11. Is there any evidence of image or data manipulation in this paper? E.g., using the same image to describe different results by re-labelling, rotating, changing the colours or contrast of the image, or cropping the image differently.

a) Yes, there was evidence of image or data manipulation in the paper.
b) No, there was no evidence of image or data manipulation in the paper.

11.1 If there was evidence of image or data manipulation, what specific alterations were identified?

12. Determine how reliable this paper is and produce a paper reliability score out of 5.

5) No issues detected.
4) Some noticeable issues, such as a few minor contradictions, or an identifiable (but not severe) conflict of interest.
3) Integrity is reasonable but weakened by repeated minor contradictions, a few irrelevant citations, or questionable choices in data presentation.
2) The paper contains major contradictions, numerous irrelevant references, and/or noticeable conflicts of interest that raise concerns about bias.
1) The paper is entirely unreliable due to extensive issues, including irrelevant citations, extreme bias from major conflicts of interest, multiple major contradictions, and/or strong indications of data or image manipulation.

Summary of Paper Contents

13. What was the aim of the study?

14. What was the hypothesis of the study?

15. Provide a comprehensive, bullet-point summary detailing the demographic characteristics of the study sample, specifically the distribution of age, sex, ethnicity, and economic status.

16. Provide a comprehensive, bullet-point summary detailing the cancer types and subtypes included in the study sample, along with the quantity of each. Specify histological subtypes, grades, stages, receptor status, and any other relevant characteristics.

17. Based on the sample demographics and the country in which this study was performed, are the findings of this study applicable to African populations in general?

a) Yes, the findings are applicable as populations across Africa share similar genetic, socioeconomic, and/or environmental conditions.
b) Partially applicable, but caution should be exercised due to differences in genetic, socioeconomic, and/or environmental conditions.
c) No, the findings cannot be applied to African populations due to significant contextual differences.
d) Applicability cannot be determined without further information about the study's sample.

18. List the most important factor(s) in the methodology that influence(s) the strength of the study's findings.

19. According to the paper, do the relevant results of this research support any other paper/s published prior to it?

a) Yes, this paper's findings support previous research.
b) No, this paper's approach is novel and thus its results do not support or contradict previous findings.
c) No, this paper's findings contradict what has previous been found.
d) Some of the findings support previous research, while other findings contradict previous research.

20. List all the papers referenced in this article that may assist in determining whether it is plausible that HMTV/MMTV-like virus can cause breast cancer in humans. Only include papers that would be classified as relevant as per the original classification criteria.

Strength of Evidence

21. If applicable, was the data collected with probability sampling (random selection)?

a) Yes, random selection was used.
b) No, random selection was not used when it should have been.
c) It is unclear whether random selection was used.
d) N/A, random selection is not possible with this study design.

22. If applicable, was the sample size for cases - and, where relevant, controls - sufficient to support the paper's conclusions?

a) Yes, the sample size was sufficient.
b) The sample size was smaller than ideal, yet sufficiently large that, combined with strong statistical findings, the conclusions remain credible.
c) No, the sample size was insufficient to support the conclusions.
d) N/A, no sample used in this study.

23. What is the statistical significance of the paper's relevant findings?

24. Was the statistical analysis appropriate in this paper?

a) Yes, all statistical analysis was appropriate.
b) No, the statistical analysis was inappropriate.
c) It is unclear what statistical analysis was performed, making it difficult to determine whether it was appropriate.

25. Are there any obvious errors in the statistical analysis or presentation of the paper's results?

a) Yes, there was an error in the paper's statistical analysis or presentation of results.
b) No, there were no errors in the paper's statistical analysis or presentation of results.

25.1 If there was an error in the statistical analysis or presentation of the results, what was it?

26. Are there any limitations to using this research as evidence for or against a plausible causative relationship between HMTV/MMTV-like virus and breast cancer?

a) Yes, there are limitations.
b) No, there are no limitations.

26.1 If there are limitations, what are they?

27. Assess the strength of evidence provided by this paper and assign a score from 1 to 5, with 1 representing severely weak / no credible evidence and 5 representing very strong evidence.

5) Very strong evidence
4) Strong evidence
3) Acceptable evidence
2) Weak evidence
1) Severely weak evidence/no credible evidence

Does this paper help fulfil or disprove the microbial oncogenesis criteria?

28. Determine if this paper helps fulfil the following listed 10 microbial oncogenicity criteria for the MMTV-like virus/HMTV and breast cancer pair. Give 1 point for each criterion the paper supports or clearly fulfils, 0 for a criterion that is either not examined by the paper or remains uncertain at the end of the paper, and -1 for any criterion that is disproved by this paper. Briefly explain the reasoning for each point or lack thereof given. Finally, determine the cumulative score from these criteria points, allocated as the causal criteria score. When answering these questions, consider the paper reliability score (Q12) and the strength of evidence score (Q30).

28.1 Epidemiologic Association: Consistent association of a microbe (or a combination of microbes) with a specific cancer type within a population (considering demographics and host factors) of humans (or across all populations), especially when compared to people within the same population(s) without cancer.

a. Tools to consider:
   i. Case-control and Cohort studies
   ii. Histopathology, immunochemistry, PCR

iii. Genomics and mass spectrometry
iv. Strong association when consistent findings across different geographical regions and consistent risk ratios in case-control or cohort studies

28.1.1 Indicate whether the paper supports, refutes, does not investigate, or leaves this criterion uncertain.

a) 0, the paper did not examine this criterion.
b) 0, the paper did investigate this criterion, but the findings are either inconclusive or uncertainty remains due to concerns about the study's reliability or strength of evidence.
c) 1, the paper provides evidence that supports this criterion.
d) -1, the paper provides evidence that refutes this criterion.

28.1.2 If this criterion was supported, refuted, or remains uncertain, select all applicable explanations below that describe why.

a) Insufficient power
b) Inappropriate methodology/tools
c) Concerns regarding paper reliability
d) Error/s in the statistical analysis
e) Sufficient power
f) Appropriate methodology/tools
g) Other (specify)

28.1.3 If you selected other, please specify.

28.1.4 State which kind of evidence is provided according to the tools to consider categories and identify the specific finding/s that fulfil or refute this criterion.

28.2 Histopathologic association: Consistent detection of the microbe (virus, bacterium, fungus, or parasite) in cancer tissues compared to healthy controls

a. Tools to consider:
   i. In Vitro assays (PCR, FACS, imaging)
   ii. Histopathology, immunochemistry, PCR
   iii. Genomics and mass spectrometry

28.2.1 Indicate whether the paper supports, refutes, does not investigate, or leaves this criterion uncertain.

a) 0, the paper did not examine this criterion.
b) 0, the paper did investigate this criterion, but the findings are either inconclusive or uncertainty remains due to concerns about the study's reliability or strength of evidence.
c) 1, the paper provides evidence that supports this criterion.
d) -1, the paper provides evidence that refutes this criterion.

28.2.2 If this criterion was supported, refuted, or remains uncertain, select all applicable explanations below that describe why.

a) Insufficient power
b) Inappropriate methodology/tools

c) Concerns regarding paper reliability
d) Error/s in the statistical analysis
e) Sufficient power
f) Appropriate methodology/tools
g) Other (specify)

28.2.3 If you selected other, please specify.

28.2.4 State which kind of evidence is provided according to the tools to consider categories and identify the specific finding/s that fulfil or refute this criterion.

28.3 Temporal association: Evidence that infection precedes onset of cancer

a. Tools to consider:
    i. Longitudinal (long term) studies

28.3.1 Indicate whether the paper supports, refutes, does not investigate, or leaves this criterion uncertain.

a) 0, the paper did not examine this criterion.
b) 0, the paper did investigate this criterion, but the findings are either inconclusive or uncertainty remains due to concerns about the study's reliability or strength of evidence.
c) 1, the paper provides evidence that supports this criterion.
d) -1, the paper provides evidence that refutes this criterion.

28.3.2 If this criterion was supported, refuted, or remains uncertain, select all applicable explanations below that describe why.

a) Insufficient power
b) Inappropriate methodology/tools
c) Concerns regarding paper reliability
d) Error/s in the statistical analysis
e) Sufficient power
f) Appropriate methodology/tools
g) Other (specify)

28.3.3 If you selected other, please specify.

28.3.4 Identify the specific finding/s that fulfil or refute this criterion.

28.4 Experimental evidence of facilitation of oncogenesis: Induction of cancer/precancerous changes upon introduction of the isolated microbe into appropriate models. Does oncogenic cellular transformation occur when a microbe is introduced into an animal (ideally a primate or other human-representative) model or tissue model?

a. Tools to consider:
    i. 3D biosystems (organoids) or animal models

28.4.1 Indicate whether the paper supports, refutes, does not investigate, or leaves this criterion uncertain.

a) 0, the paper did not examine this criterion.

b) 0, the paper did investigate this criterion, but the findings are either inconclusive or uncertainty remains due to concerns about the study's reliability or strength of evidence.
c) 1, the paper provides evidence that supports this criterion.
d) -1, the paper provides evidence that refutes this criterion.

28.4.2 If this criterion was supported, refuted, or remains uncertain, select all applicable explanations below that describe why.

a) Insufficient power
b) Inappropriate methodology/tools
c) Concerns regarding paper reliability
d) Error/s in the statistical analysis
e) Sufficient power
f) Appropriate methodology/tools
g) Other (specify)

28.4.3 If you selected other, please specify.

28.4.4 Identify the specific finding/s that fulfil or refute this criterion.

28.5 Molecular and Multi-omics evidence for interaction:

i. Epigenetics (e.g. DNA methylation changes in H. pylori or HPV E6 oncogene resulting in P53 degradation), as well as mutational signatures
ii. Mutation effects (stimulating cellular mutations promoting cancer)
iii. Stimulating immune evasion mechanisms reducing immunosurveillance for emerging cancer cells
iv. Integration of microbial DNA into host genome

a. Tools to consider:
   i. in vitro assays, organoids, multi-omics assessments including:
      a. **Molecular Evidence**
         i. For bacteria: Identification of toxins, effector proteins, or metabolites that alter cellular signalling
         ii. For viruses: Characterization of viral oncoproteins or insertional mutagenesis
         iii. For fungi: Demonstration of mycotoxins or immune-modulating molecules with carcinogenic potential
         iv. For parasites: Identification of chronic inflammatory responses or direct tissue damage mechanisms
         v. Evidence of microbial interference with DNA repair, cell cycle regulation, apoptosis, or immune surveillance
      b. **Genomic evidence:**
         i. Microbial DNA sequences or integration sites in tumor genome;
         ii. Host genetic susceptibility factors that enhance oncogenic potential of specific microbes, including demonstration of how microbial exposure can alter (or promote) known host genetic risk factors for cancer.
         iii. Demonstratable facilitation of cancer-associated mutational signatures.
      c. **Transcriptomic evidence:**
         i. Expression of microbial genes or altered host gene expression profiles

d. **Proteomic evidence:**
   i. Detection of microbial proteins or altered host protein responses
e. **Metabolomic evidence:**
   i. Microbial metabolites or altered host metabolic pathways
f. **Microbiome analyses:**
   i. Consistent dysbiosis patterns associated with specific cancers

28.5.1 Indicate whether the paper supports, refutes, does not investigate, or leaves this criterion uncertain.

a) 0, the paper did not examine this criterion.
b) 0, the paper did investigate this criterion, but the findings are either inconclusive or uncertainty remains due to concerns about the study's reliability or strength of evidence.
c) 1, the paper provides evidence that supports this criterion.
d) -1, the paper provides evidence that refutes this criterion.

28.5.2 If this criterion was supported, refuted, or remains uncertain, select all applicable explanations below that describe why.

a) Insufficient power
b) Inappropriate methodology/tools
c) Concerns regarding paper reliability
d) Error/s in the statistical analysis
e) Sufficient power
f) Appropriate methodology/tools
g) Other (specify)

28.5.3 If you selected other, please specify.

28.5.4 State which kind of evidence is provided according to the tools to consider categories and identify the specific finding/s that fulfil or refute this criterion.

28.6 Prevention: Does preventing the infection (or removing exposure to the microbe) reduce cancer or evidence of oncogenesis

a. Tools to consider:
   i. Clinical trials
   ii. Relevant animal models
   iii. For viruses: Reduced cancer incidence following vaccination (e.g., HPV, HBV)
   iv. For bacteria: Cancer prevention through antibiotic treatment or bacterial elimination
   v. For fungi: Antifungal intervention effects on precancerous lesions
   vi. For parasites: Impact of antiparasitic treatment on cancer development
   vii. Prevention trials showing reduced cancer incidence after targeting the microbe

28.6.1 Indicate whether the paper supports, refutes, does not investigate, or leaves this criterion uncertain.

a) 0, the paper did not examine this criterion.
b) 0, the paper did investigate this criterion, but the findings are either inconclusive or uncertainty remains due to concerns about the study's reliability or strength of evidence.

c) 1, the paper provides evidence that supports this criterion.
d) -1, the paper provides evidence that refutes this criterion.

28.6.2 If this criterion was supported, refuted, or remains uncertain, select all applicable explanations below that describe why.

a) Insufficient power
b) Inappropriate methodology/tools
c) Concerns regarding paper reliability
d) Error/s in the statistical analysis
e) Sufficient power
f) Appropriate methodology/tools
g) Other (specify)

28.6.3 If you selected other, please specify.

28.6.4 State which kind of evidence is provided according to the tools to consider categories and identify the specific finding/s that fulfil or refute this criterion.

28.7 Reproducibility and Validation: Confirmation across independent laboratories, optimally using varied methodologies or models. Replication in diverse human populations and geographic settings. Concordance between in vitro, animal, and human studies

28.7.1 Indicate whether the paper supports, refutes, does not investigate, or leaves this criterion uncertain.

a) 0, the paper did not examine this criterion.
b) 0, the paper did investigate this criterion, but the findings are either inconclusive or uncertainty remains due to concerns about the study's reliability or strength of evidence.
c) 1, the paper provides evidence that supports this criterion.
d) -1, the paper provides evidence that refutes this criterion.

28.7.2 If this criterion was supported, refuted, or remains uncertain, select all applicable explanations below that describe why.

a) Insufficient power
b) Inappropriate methodology/tools
c) Concerns regarding paper reliability
d) Error/s in the statistical analysis
e) Sufficient power
f) Appropriate methodology/tools
g) Other (specify)

28.7.3 If you selected other, please specify.

28.7.4 Identify the specific finding/s that fulfil or refute this criterion.

28.8 Dose Response relationship: Relationship of severity or chronicity of the presumed offending infection with cancer initiation and severity in human longitudinal studies or in experimental models demonstrate the association of infectious dose with development of cancer

a. Tools to consider:
    i. Organoids, animal models, or clinico-epidemiologic longitudinal studies

28.8.1 Indicate whether the paper supports, refutes, does not investigate, or leaves this criterion uncertain.

a) 0, the paper did not examine this criterion.
b) 0, the paper did investigate this criterion, but the findings are either inconclusive or uncertainty remains due to concerns about the study's reliability or strength of evidence.
c) 1, the paper provides evidence that supports this criterion.
d) -1, the paper provides evidence that refutes this criterion.

28.8.2 If this criterion was supported, refuted, or remains uncertain, select all applicable explanations below that describe why.

a) Insufficient power
b) Inappropriate methodology/tools
c) Concerns regarding paper reliability
d) Error/s in the statistical analysis
e) Sufficient power
f) Appropriate methodology/tools
g) Other (specify)

28.8.3 If you selected other, please specify.

28.8.4 State which kind of evidence is provided according to the tools to consider categories and identify the specific finding/s that fulfil or refute this criterion.

28.9 Plausibility: Are there plausible mechanisms for considering a potential role for a microbe

a. Tools to consider:
    i. Knowledge-based; i.e. understanding the physiology of microbial interaction with humans may suggest or support a role in carcinogenesis

28.9.1 Indicate whether the paper supports, refutes, does not discuss, or leaves this criterion uncertain.

a) 0, the paper does not suggest or discuss any plausible mechanisms for the MCP.
b) 0, the paper suggests a possible mechanism, but it is weakly justified, inconsistent with existing knowledge, or supported by insufficient evidence from the study.
c) 1, the paper presents a plausible mechanism, either consistent with existing knowledge of cancer biology or microbial pathogenesis or supported by credible evidence produced by the present study.
d) -1, the paper presents reasoning or mechanisms that argue against a plausible role for the microbe in carcinogenesis.

28.9.2 If this criterion was supported, refuted, or remains uncertain, select all applicable explanations below that describe why.

a) Insufficient power
b) Inappropriate methodology/tools
c) Concerns regarding paper reliability

d) Error/s in the statistical analysis
e) Sufficient power
f) Appropriate methodology/tools
g) Other (specify)

28.9.3 If you selected other, please specify.

28.9.4 Identify the specific finding/s that fulfil or refute this criterion.

28.10 Impact of co-Factors: Microbial carcinogenesis occurs (or is accelerated) in an environment with promotive host factors, such as genetic risk, immunodeficiencies, nutritional status, and/or with environmental factors (including but not limited to known carcinogens)

a. Tools to consider:
   i. Epidemiological studies in humans
   ii. Laboratory studies
   iii. Animal models

28.10.1 Indicate whether the paper supports, refutes, does not investigate, or leaves this criterion uncertain.

a) 0, the paper did not examine this criterion.
b) 0, the paper did investigate this criterion, but the findings are either inconclusive or uncertainty remains due to concerns about the study's reliability or strength of evidence.
c) 1, the paper provides evidence that supports this criterion.
d) -1, the paper provides evidence that refutes this criterion.

28.10.2 If this criterion was supported, refuted, or remains uncertain, select all applicable explanations below that describe why.

a) Insufficient power
b) Inappropriate methodology/tools
c) Concerns regarding paper reliability
d) Error/s in the statistical analysis
e) Sufficient power
f) Appropriate methodology/tools
g) Other (specify)

28.10.3 If you selected other, please specify.

28.10.4 State which kind of evidence is provided according to the tools to consider categories and identify the specific finding/s that fulfil or refute this criterion.

29 Calculate the cumulative score from these criteria points and provide the final causal criteria score.

30 Indicate whether the paper supports, refutes, or does not impact the plausibility of HMTV/MMTV-like virus causing breast cancer.

5) Strongly supports (moderate to strong evidence)
4) Weakly supports (weak to moderate evidence)

3) Does not impact (negligible or weak evidence)
2) Weakly refutes (weak to moderate evidence)
1) Strongly refutes (moderate to strong evidence)

1.4 Abridged Extraction Template for Repeat Experiments

This version of the extraction template was used for repeat experiments (30) with GPT-5. For the second paper, question item 4.4 (microbial oncogenesis criterion “Impact of co-factors”) was replaced with the microbial oncogenesis criterion “Plausibility” question item (see question item 28.9 and 28.9.1 in the full extraction template).

You are a medical researcher analysing the literature to evaluate the plausibility of HMTV/MMTV-like virus as a causative agent of breast cancer in humans. To achieve this, carefully review the given paper and respond to the questions that follow.

Relevancy

1. How relevant is this article in determining whether it is plausible that HMTV/MMTV-like virus can cause breast cancer in humans (relevant, somewhat relevant, or irrelevant)? A relevant article would fulfil either of the following criteria: 1) gives causal evidence for whether or not HMTV/MMTV-like virus or its species/family of microbes cause breast cancer or other cancers either in humans or animal models, or 2) contains a theoretical model for how HMTV/MMTV-like virus might cause breast cancer, irrespective of whether there is experimental evidence. A somewhat relevant article would fulfil either of the following criteria: 1) mentions both HMTV/MMTV-like virus and breast cancer, but does not give any causal arguments or evidence for HMTV/MMTV-like virus causing breast cancer, or 2) contains evidence examining whether other microbes cause breast cancer, if the findings can plausibly generalise to HMTV/MMTV-like virus and breast cancer. An irrelevant article would be a paper that does not fulfil the criteria for the relevant or somewhat relevant classifications.

a) Relevant
b) Somewhat relevant
c) Irrelevant

Strength of Evidence

2. If applicable, was the sample size for cases - and, where relevant, controls - sufficient to support the paper’s conclusions?

a) Yes, the sample size was sufficient.
b) The sample size was smaller than ideal, yet sufficiently large that, combined with strong statistical findings, the conclusions remain credible.
c) No, the sample size was insufficient to support the conclusions.
d) N/A, no sample used in this study.

3. Assess the strength of evidence provided by this paper and assign a score from 1 to 5, with 1 representing severely weak / no credible evidence and 5 representing very strong evidence.

5) Very strong evidence
4) Strong evidence
3) Acceptable evidence
2) Weak evidence
1) Severely weak evidence/no credible evidence

Does this paper help fulfil or disprove the microbial oncogenesis criteria?

4. Determine if this paper helps fulfil the following listed microbial oncogenicity criteria for the MMTV-like virus/HMTV and breast cancer pair. Give 1 point for each criterion the paper supports or clearly fulfils, 0 for a criterion that is either not examined by the paper or remains uncertain at the end of the paper, and -1 for any criterion that is disproved by this paper. When answering these questions, consider the strength of evidence score (Q3).

4.1 Epidemiologic Association: Consistent association of a microbe (or a combination of microbes) with a specific cancer type within a population (considering demographics and host factors) of humans (or across all populations), especially when compared to people within the same population(s) without cancer.

a. Tools to consider:
   i. Case-control and Cohort studies
   ii. Histopathology, immunochemistry, PCR
   iii. Genomics and mass spectrometry
   iv. Strong association when consistent findings across different geographical regions and consistent risk ratios in case-control or cohort studies

4.1.1 Indicate whether the paper supports, refutes, does not investigate, or leaves this criterion uncertain.

a) 0, the paper did not examine this criterion.
b) 0, the paper did investigate this criterion, but the findings are either inconclusive or uncertainty remains due to concerns about the study's reliability or strength of evidence.
c) 1, the paper provides evidence that supports this criterion.
d) -1, the paper provides evidence that refutes this criterion.

4.2 Histopathologic association: Consistent detection of the microbe (virus, bacterium, fungus, or parasite) in cancer tissues compared to healthy controls

a. Tools to consider:
   i. In Vitro assays (PCR, FACS, imaging)
   ii. Histopathology, immunochemistry, PCR
   iii. Genomics and mass spectrometry

4.2.1 Indicate whether the paper supports, refutes, does not investigate, or leaves this criterion uncertain.

a) 0, the paper did not examine this criterion.
b) 0, the paper did investigate this criterion, but the findings are either inconclusive or uncertainty remains due to concerns about the study's reliability or strength of evidence.
c) 1, the paper provides evidence that supports this criterion.
d) -1, the paper provides evidence that refutes this criterion.

4.3 Dose Response relationship: Relationship of severity or chronicity of the presumed offending infection with cancer initiation and severity in human longitudinal studies or in experimental models demonstrate the association of infectious dose with development of cancer

a. Tools to consider:

i. Organoids, animal models, or clinico-epidemiologic longitudinal studies

4.3.1 Indicate whether the paper supports, refutes, does not investigate, or leaves this criterion uncertain.

a) 0, the paper did not examine this criterion.
b) 0, the paper did investigate this criterion, but the findings are either inconclusive or uncertainty remains due to concerns about the study's reliability or strength of evidence.
c) 1, the paper provides evidence that supports this criterion.
d) -1, the paper provides evidence that refutes this criterion.

4.4 Impact of co-Factors: Microbial carcinogenesis occurs (or is accelerated) in an environment with promotive host factors, such as genetic risk, immunodeficiencies, nutritional status, and/or with environmental factors (including but not limited to known carcinogens)

a. Tools to consider:
   i. Epidemiological studies in humans
   ii. Laboratory studies
   iii. Animal models

4.4.1 Indicate whether the paper supports, refutes, does not investigate, or leaves this criterion uncertain.

a) 0, the paper did not examine this criterion.
b) 0, the paper did investigate this criterion, but the findings are either inconclusive or uncertainty remains due to concerns about the study's reliability or strength of evidence.
c) 1, the paper provides evidence that supports this criterion.
d) -1, the paper provides evidence that refutes this criterion.

5 Indicate whether the paper supports, refutes, or does not impact the plausibility of HMTV/MMTV-like virus causing breast cancer.

5) Strongly supports (moderate to strong evidence)
4) Weakly supports (weak to moderate evidence)
3) Does not impact (negligible or weak evidence)
2) Weakly refutes (weak to moderate evidence)
1) Strongly refutes (moderate to strong evidence)
